# Supplementary material for: CD34 and CD105 Microvessels in Resected Bone Specimen May Implicate Wound Healing in MRONJ
Source: Int J Environ Res Public Health. 2021 Oct 29;18(21):11362. doi: 10.3390/ijerph182111362 (PMC8582901; doi:10.3390/ijerph182111362)

**ID:1 CD34**

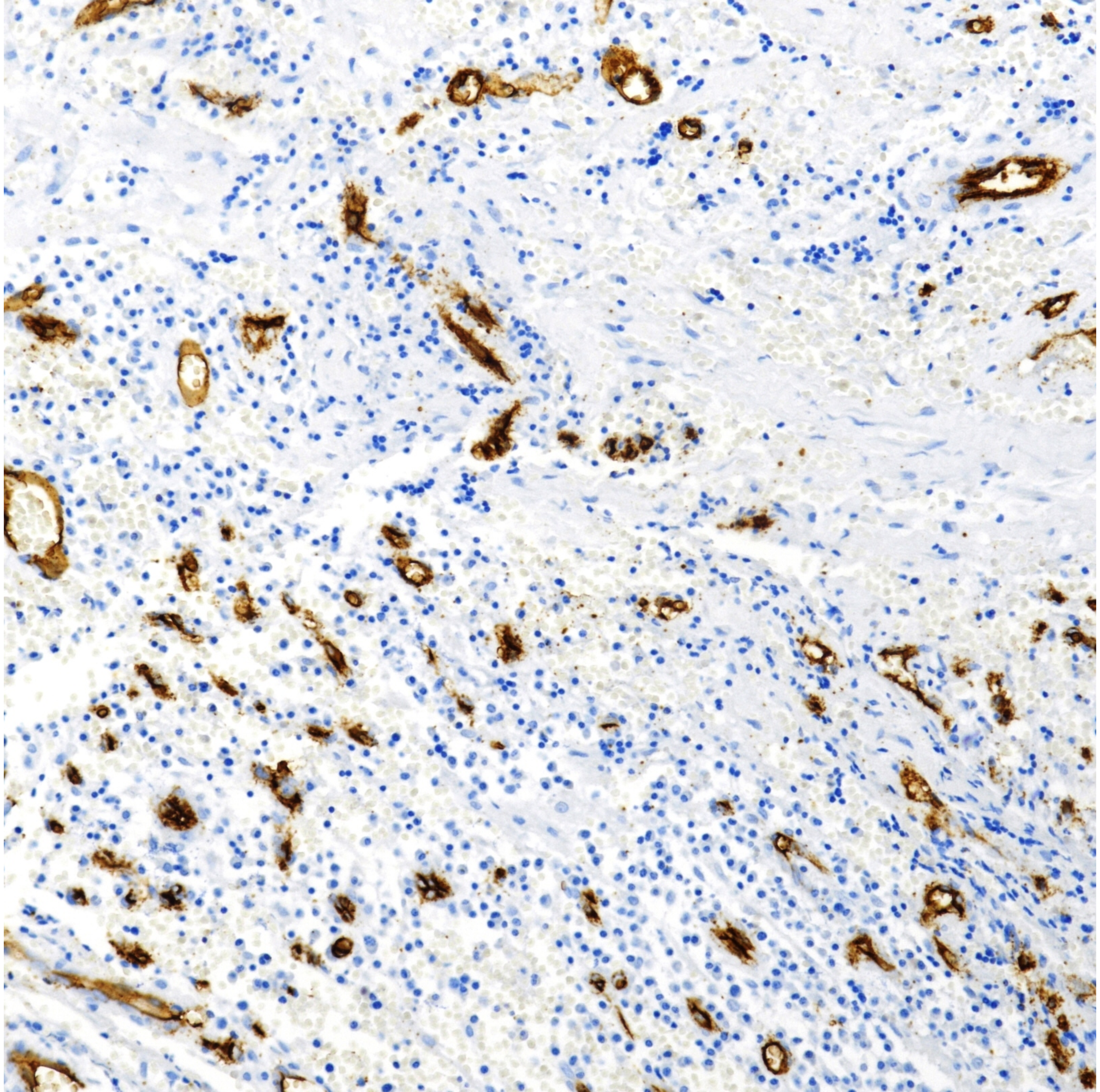

**ID:1 CD105**

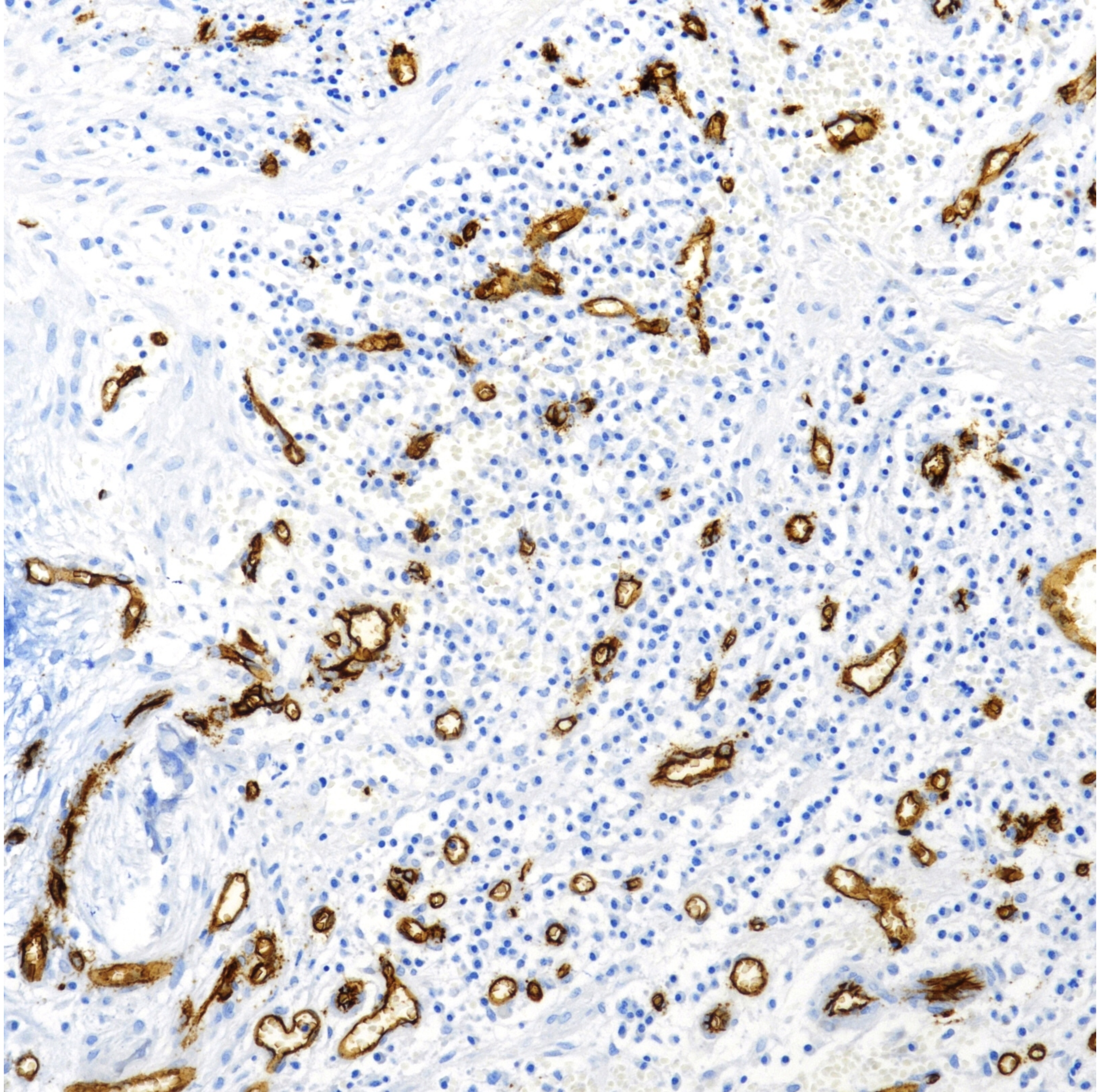

ID:2 CD34

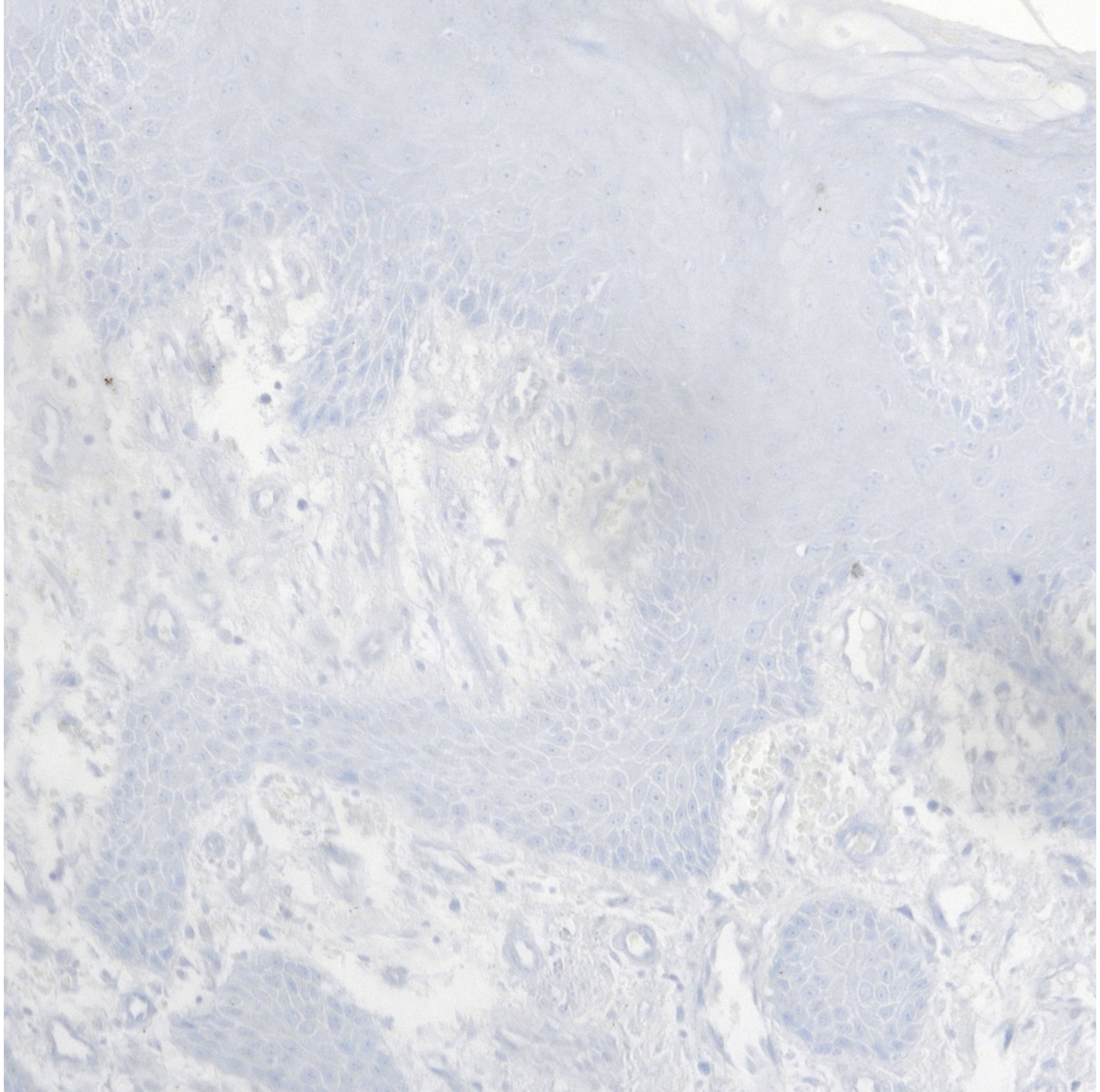

**ID:2  CD105**

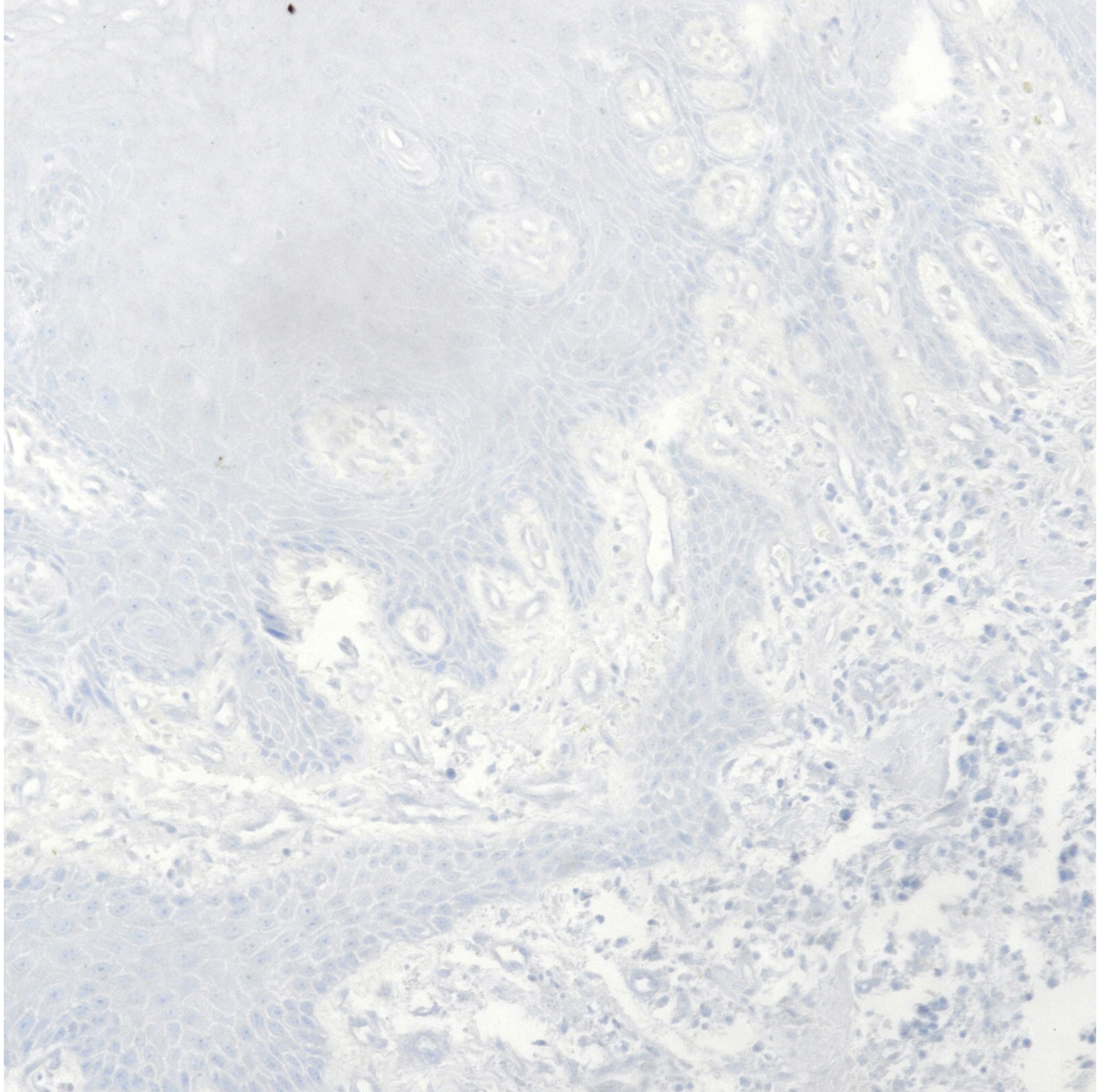

ID:3 CD34

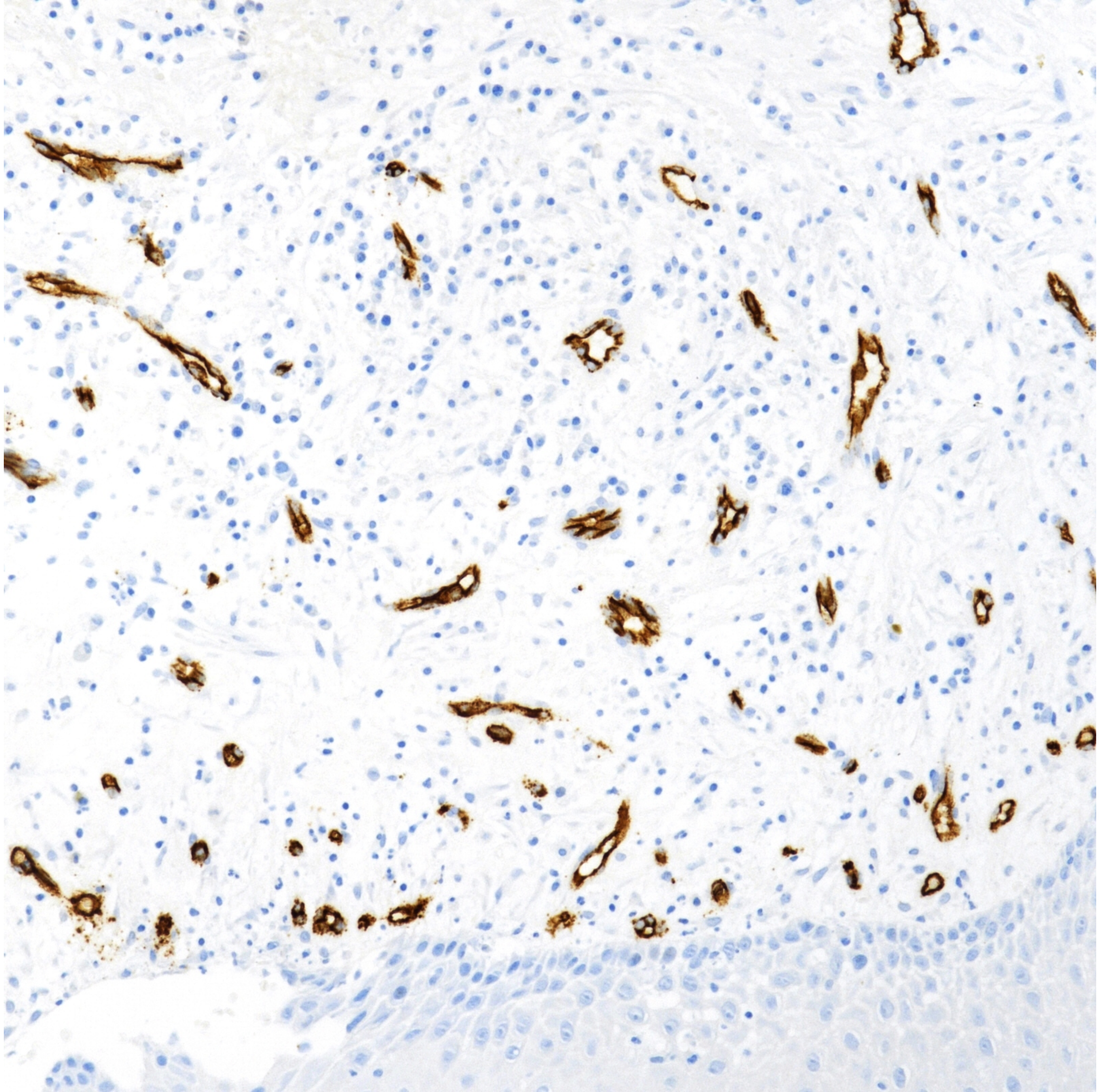

ID:3  CD105

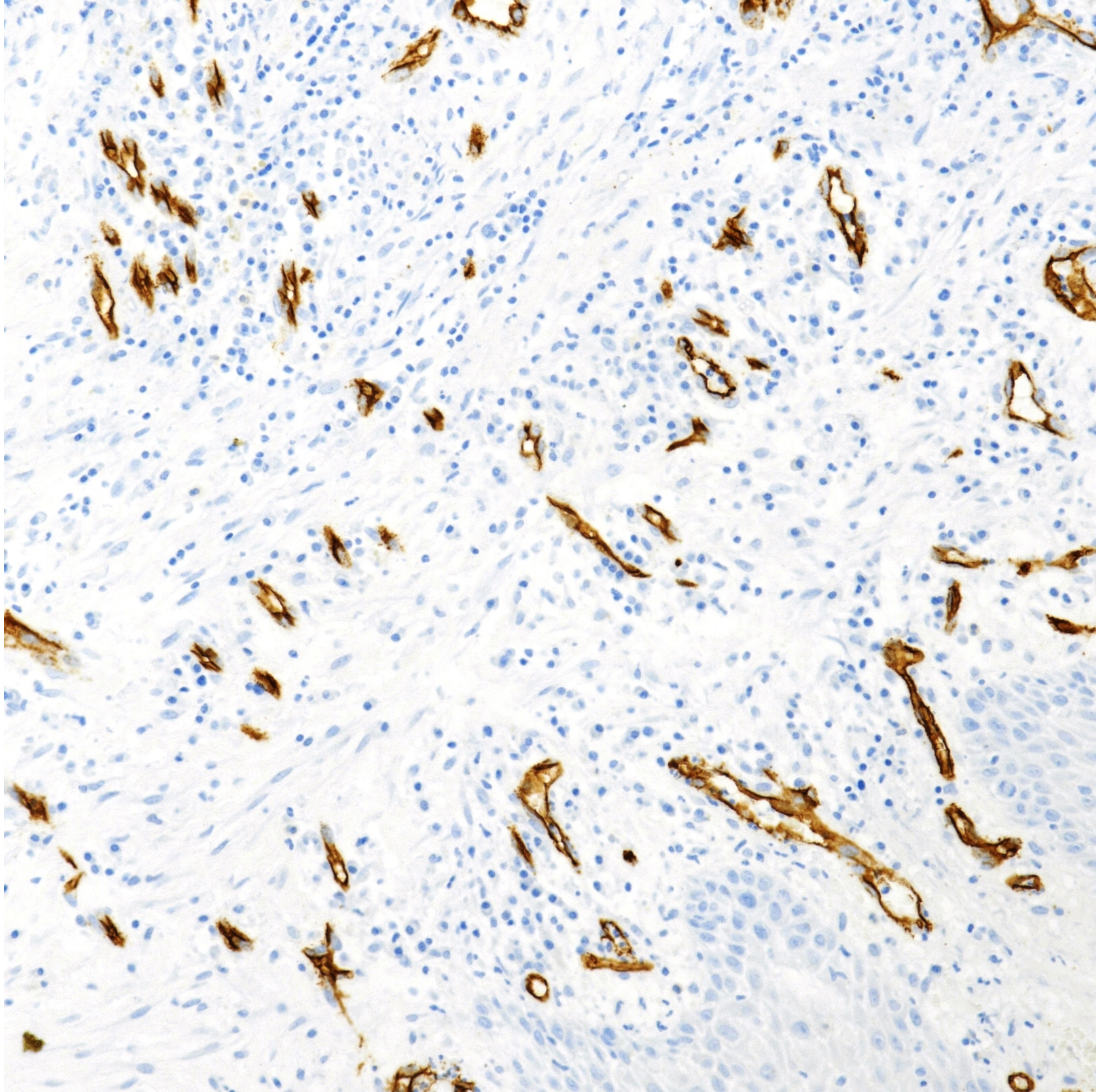

ID:4 CD34

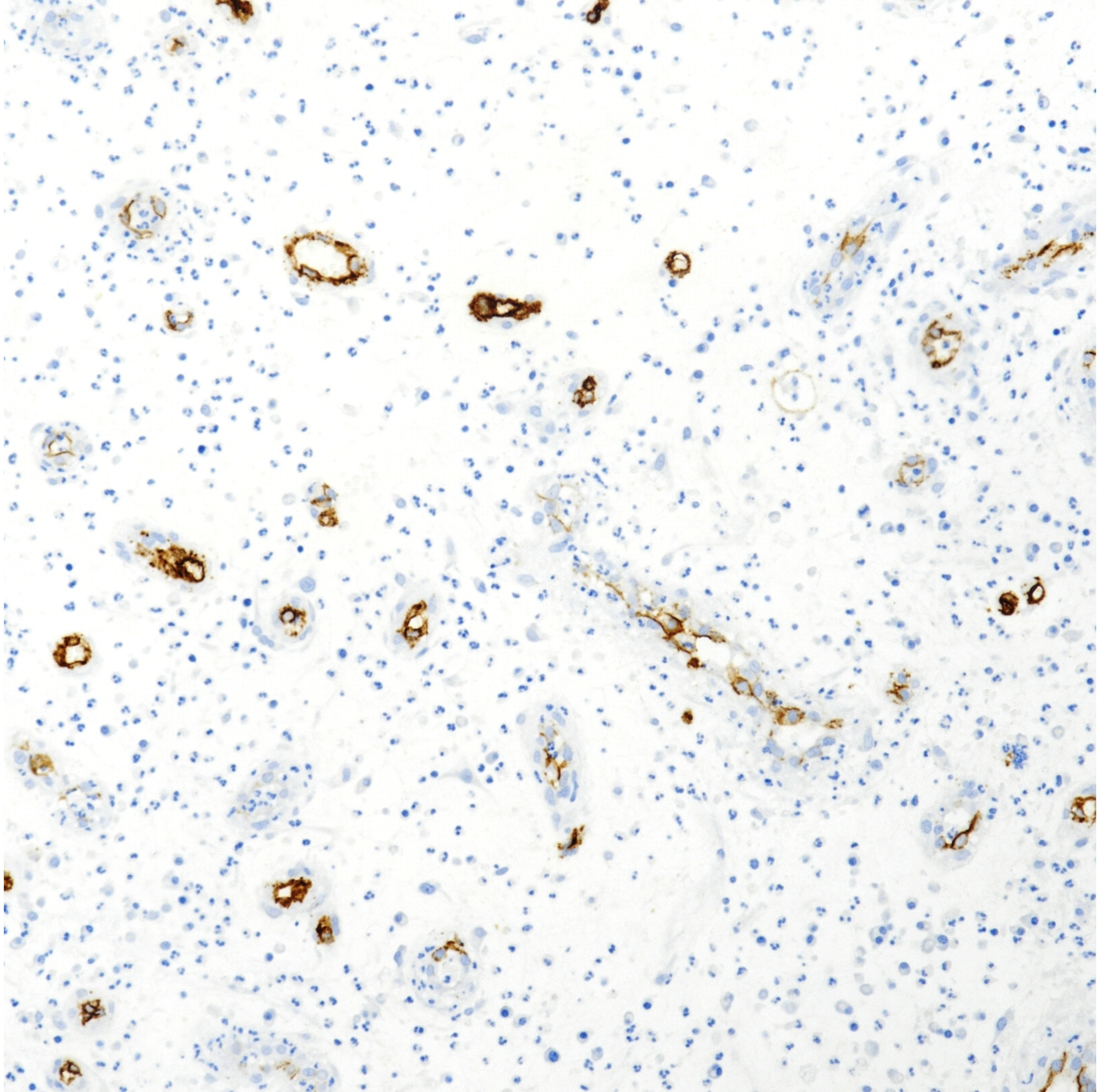

ID:4  CD105

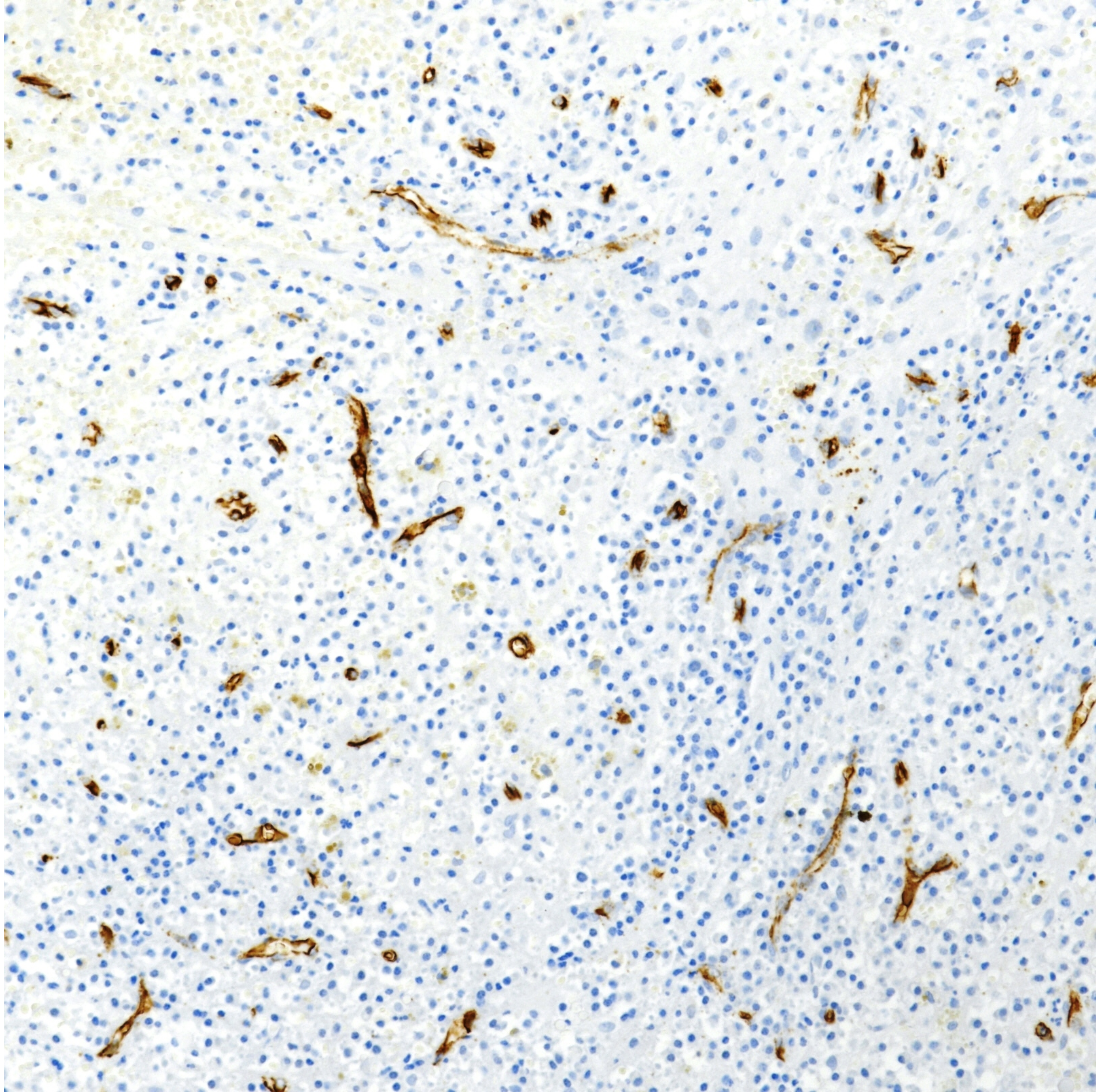

ID:5 CD34

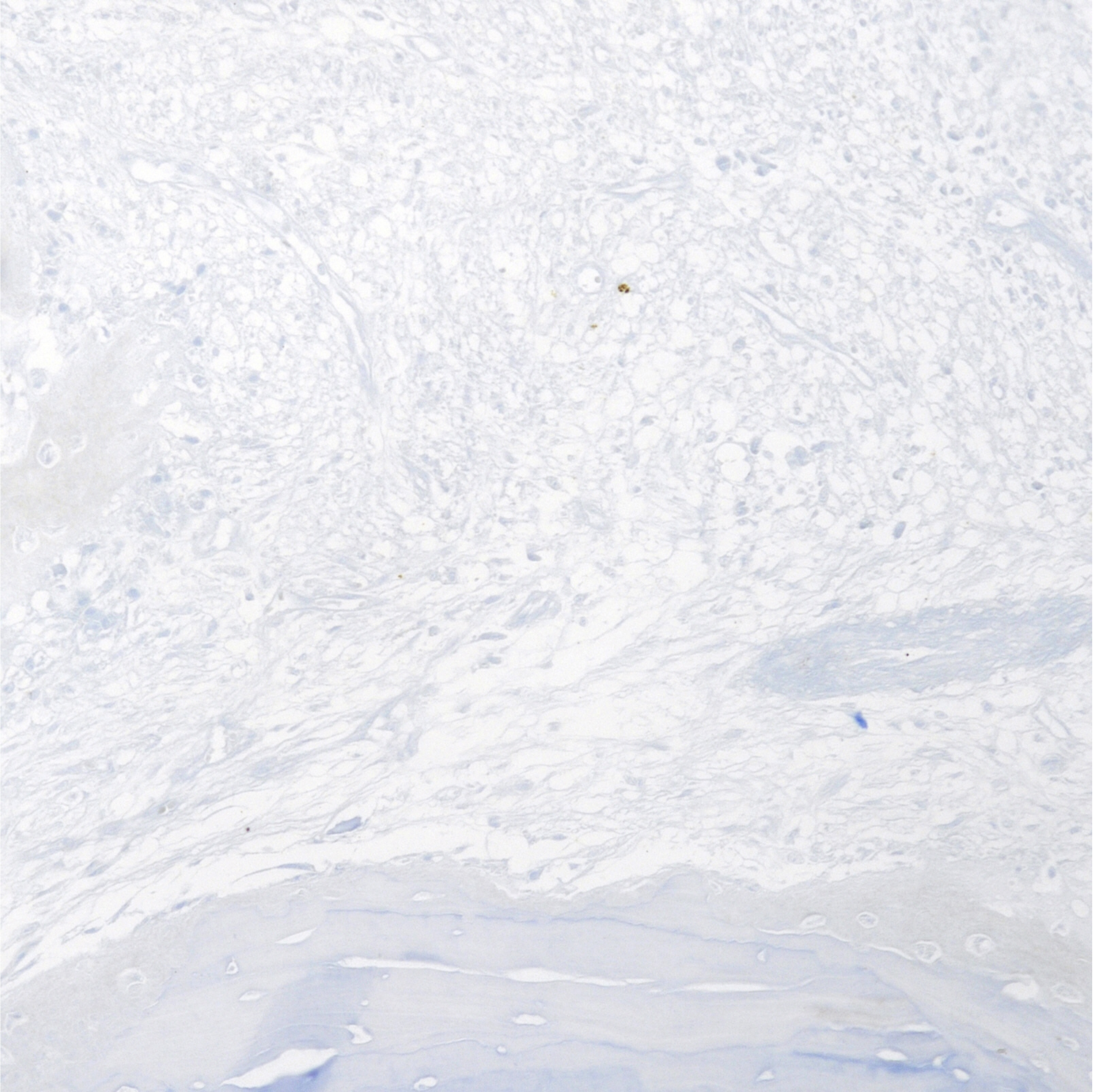

ID:5  CD105

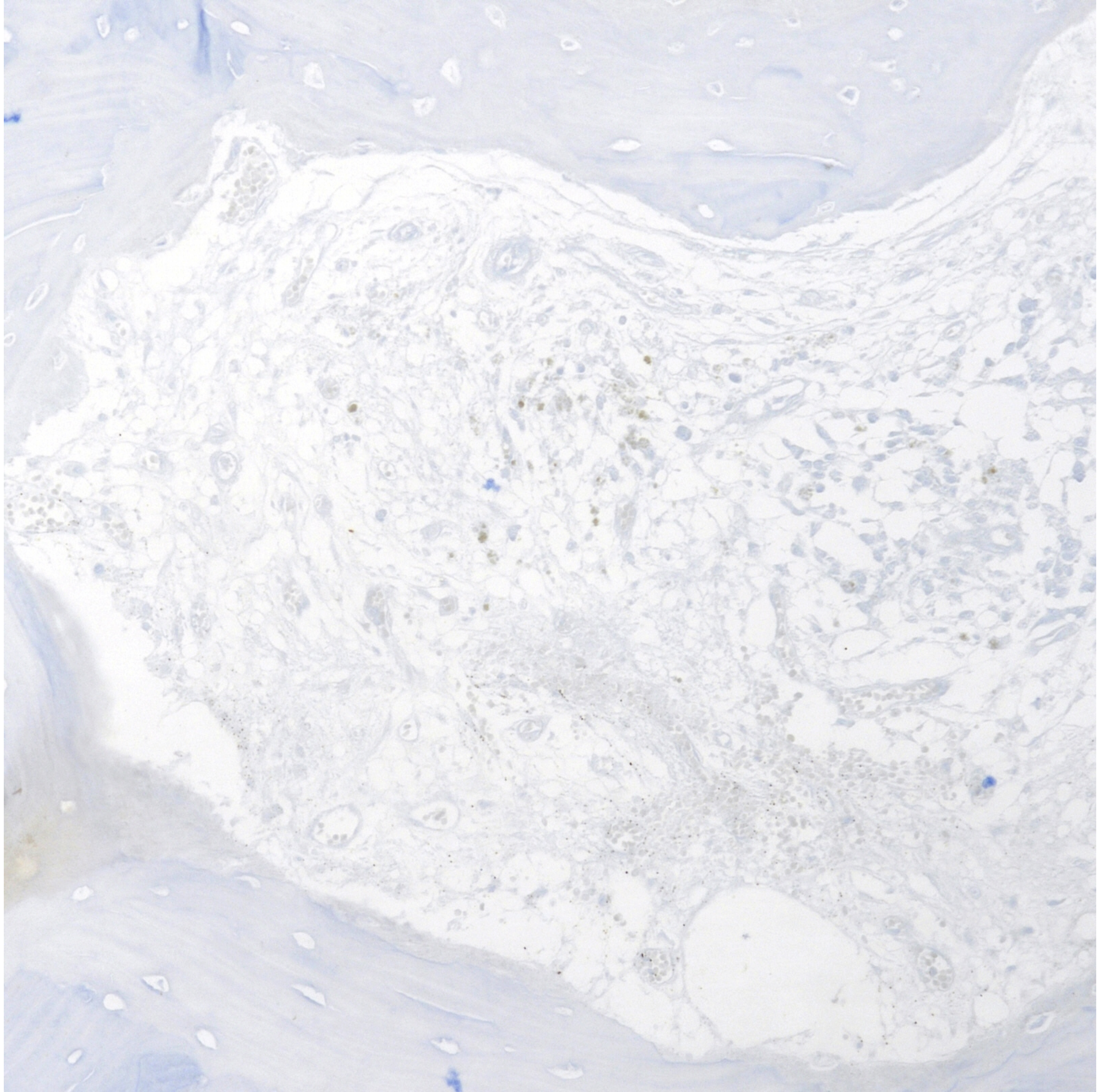

ID:6 CD34

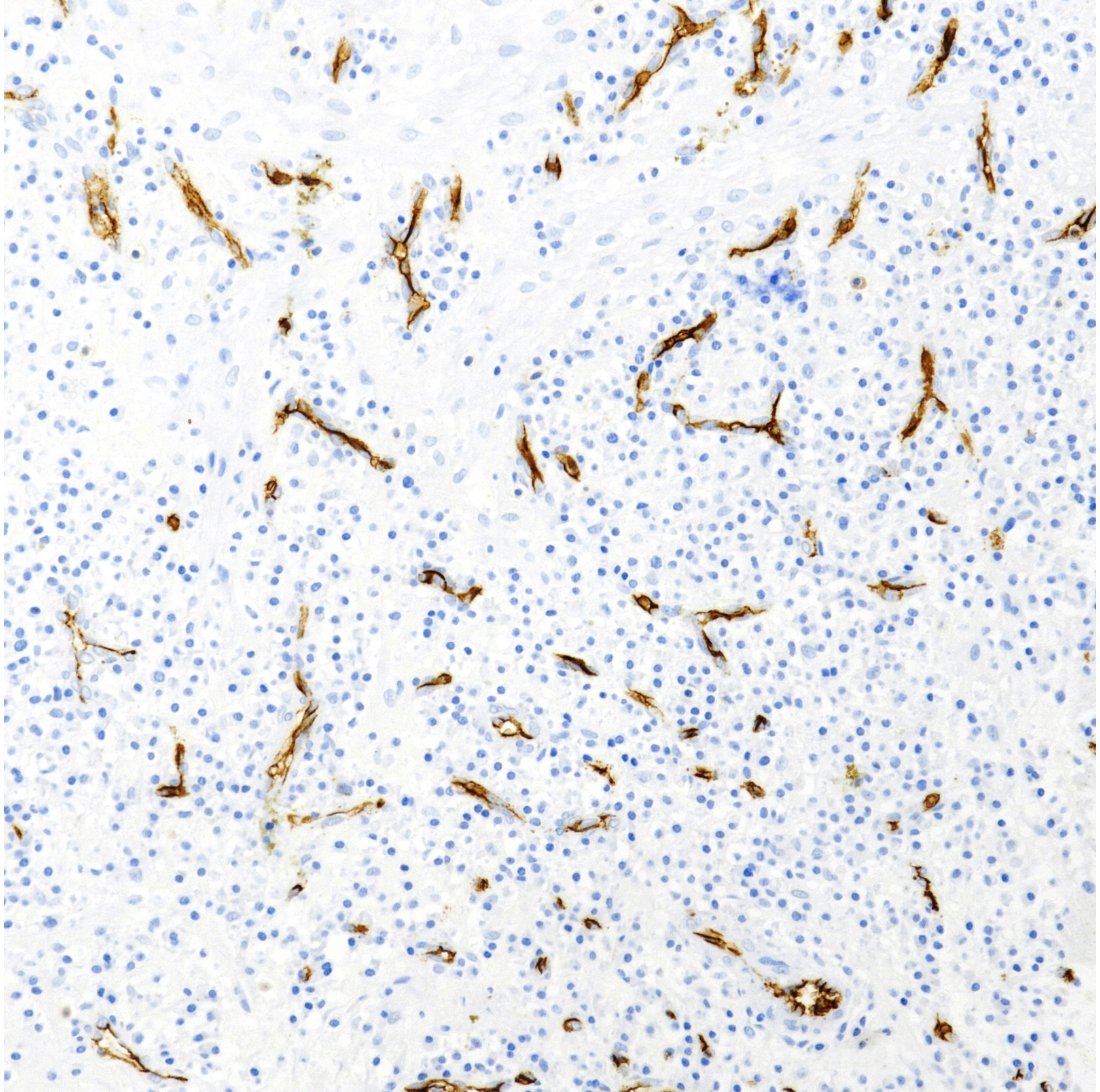

ID:6 CD105

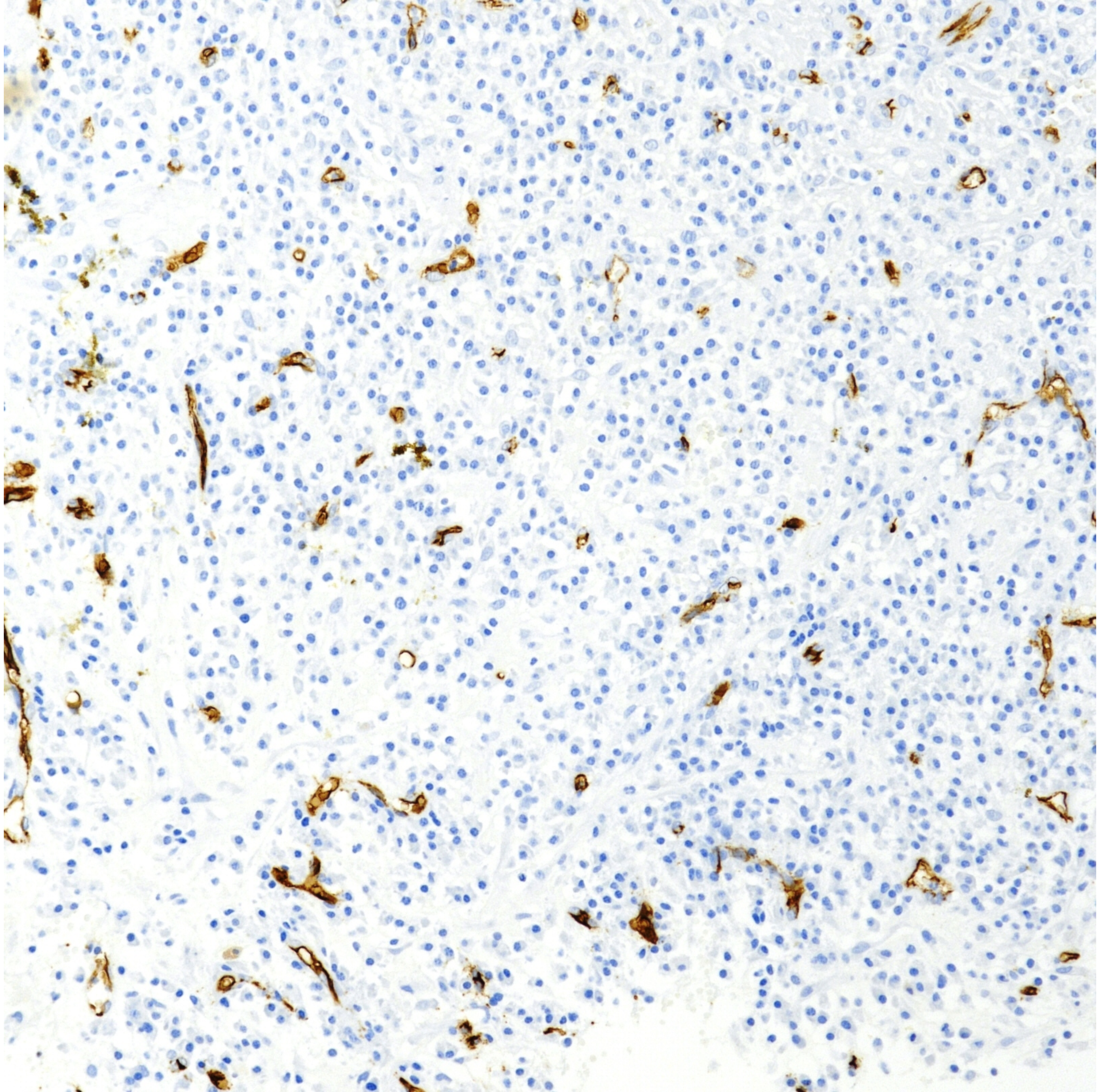

ID:7 CD34

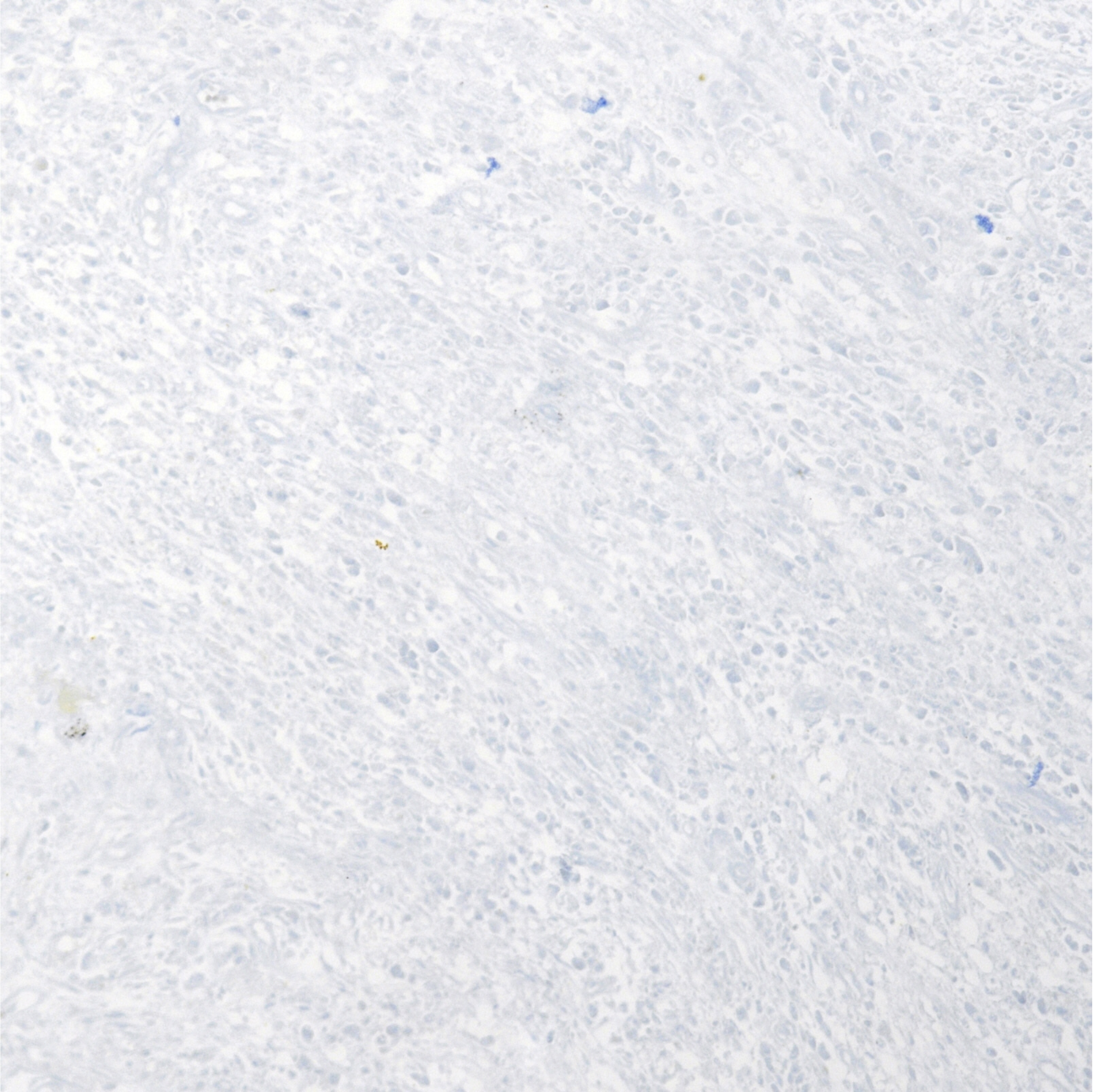

**ID:7 CD105**

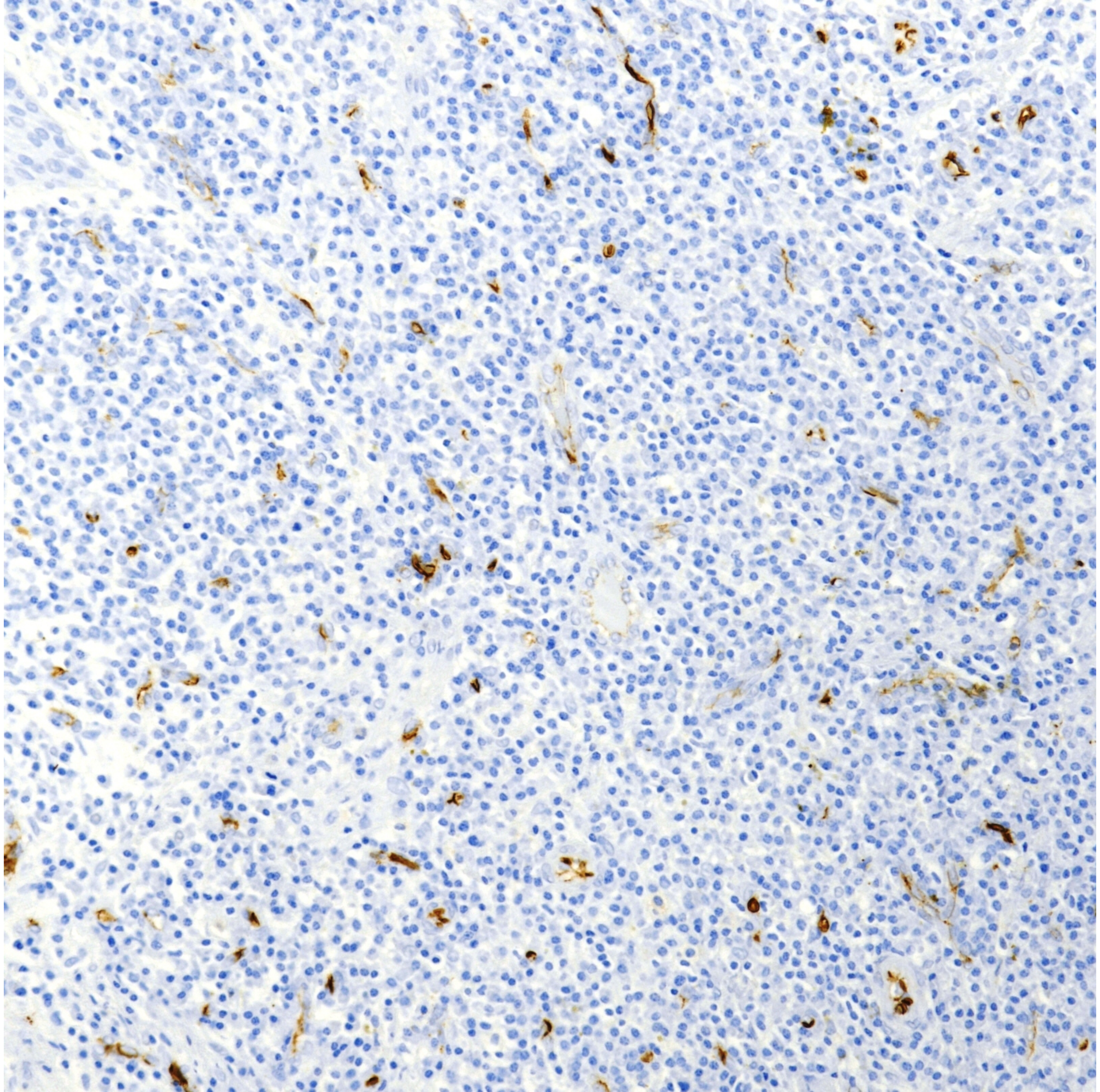

**ID:8 CD34**

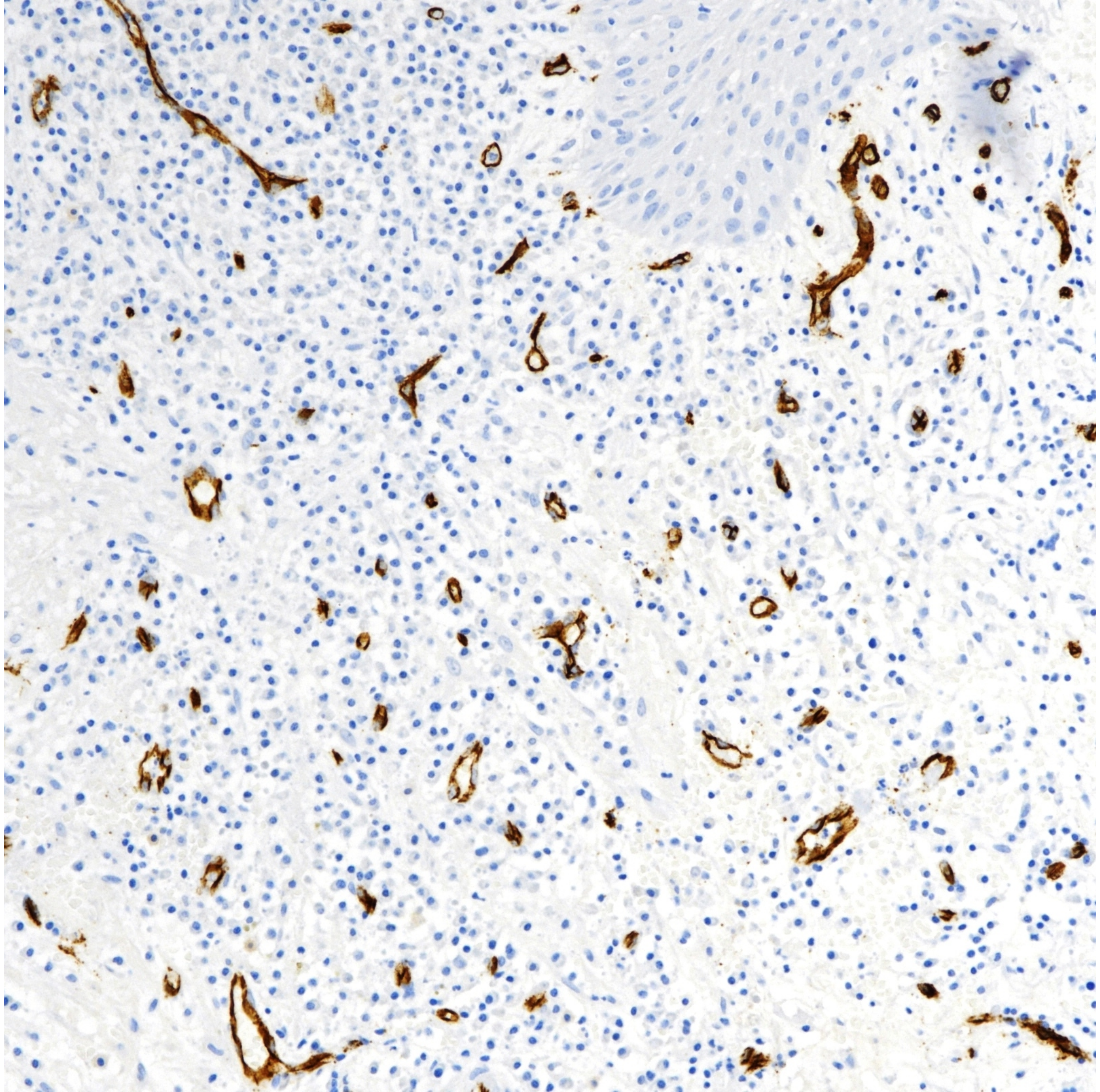

**ID:8  CD105**

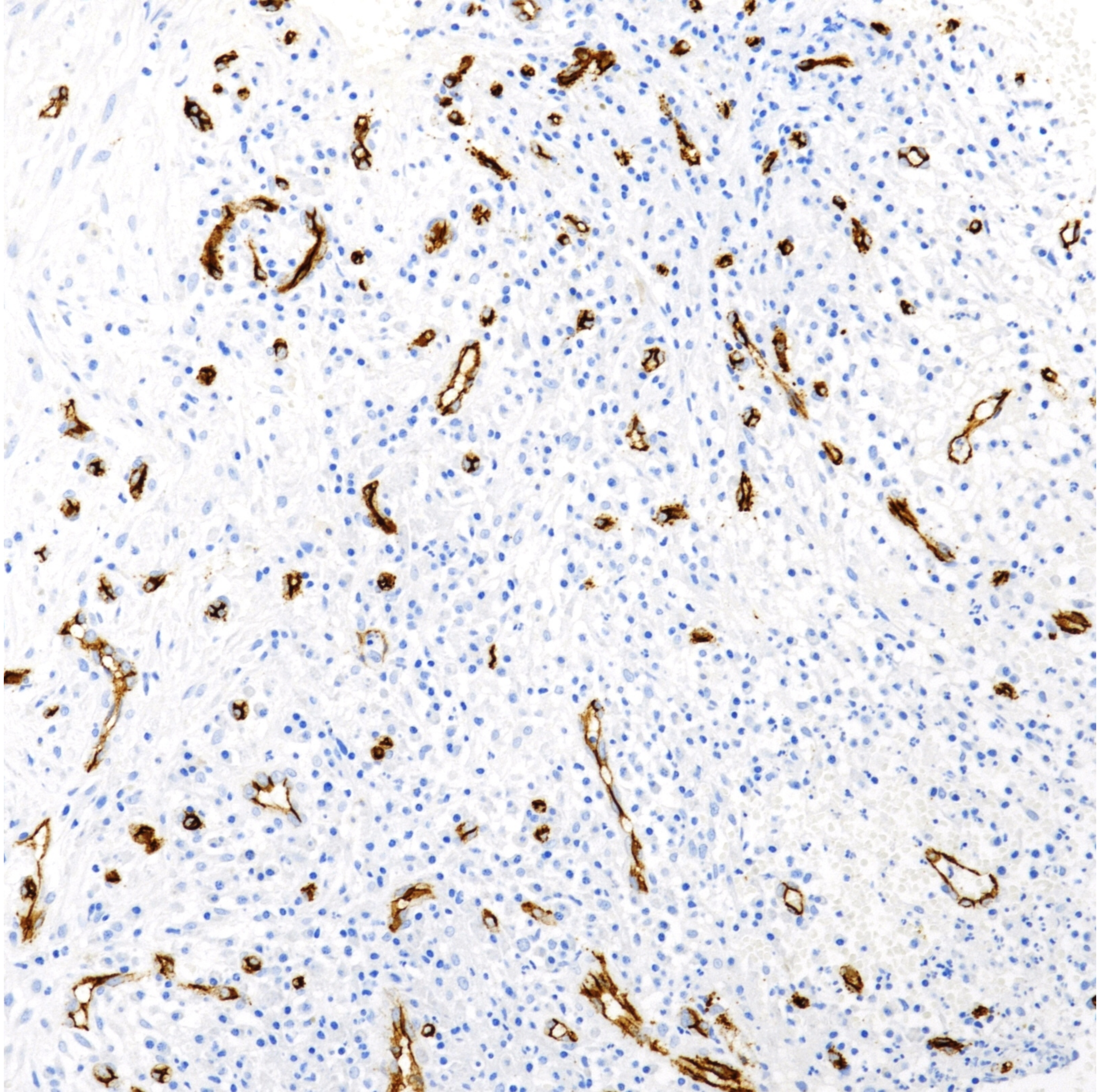

**ID:9 CD34**

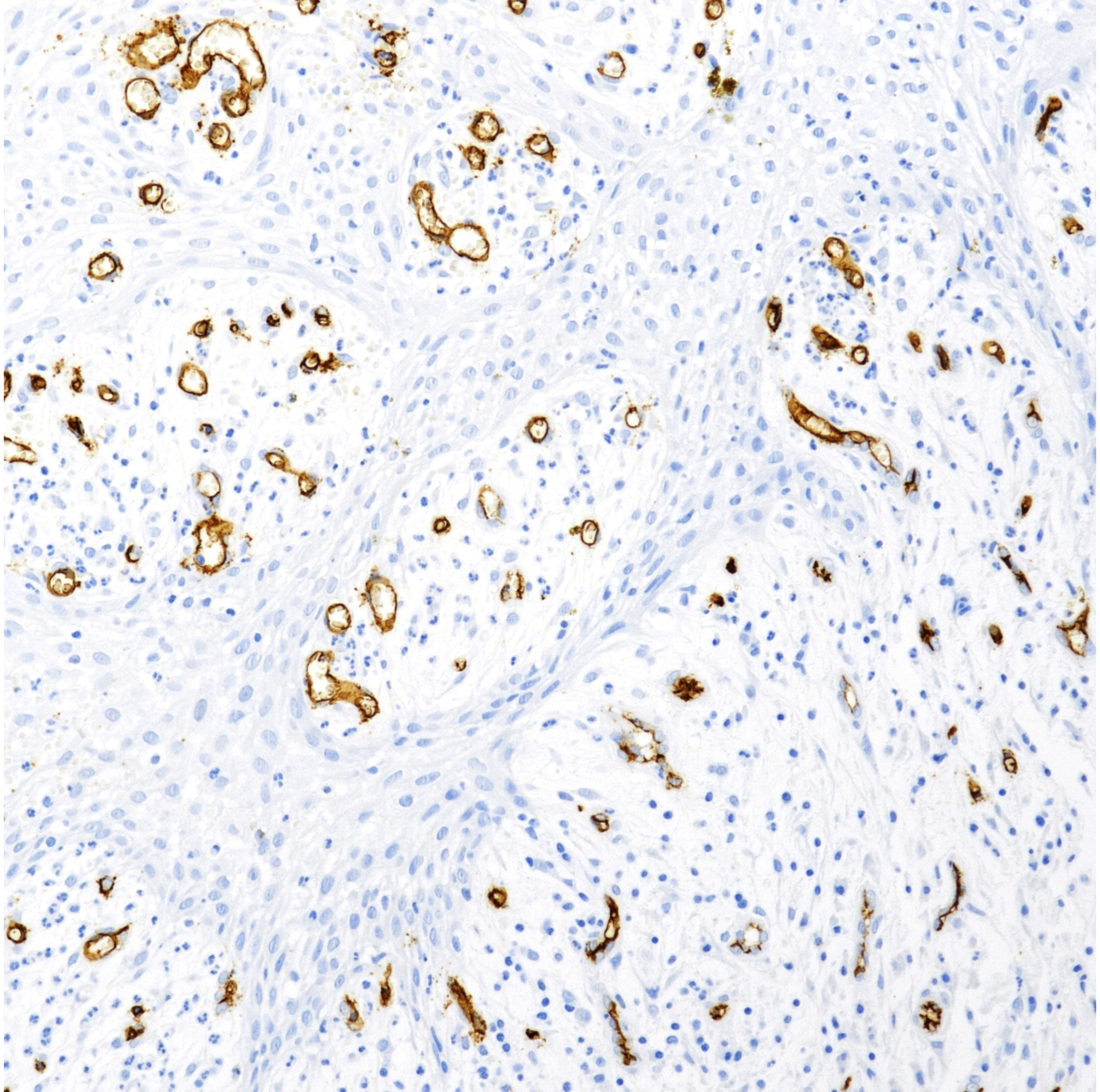

**ID:9  CD105**

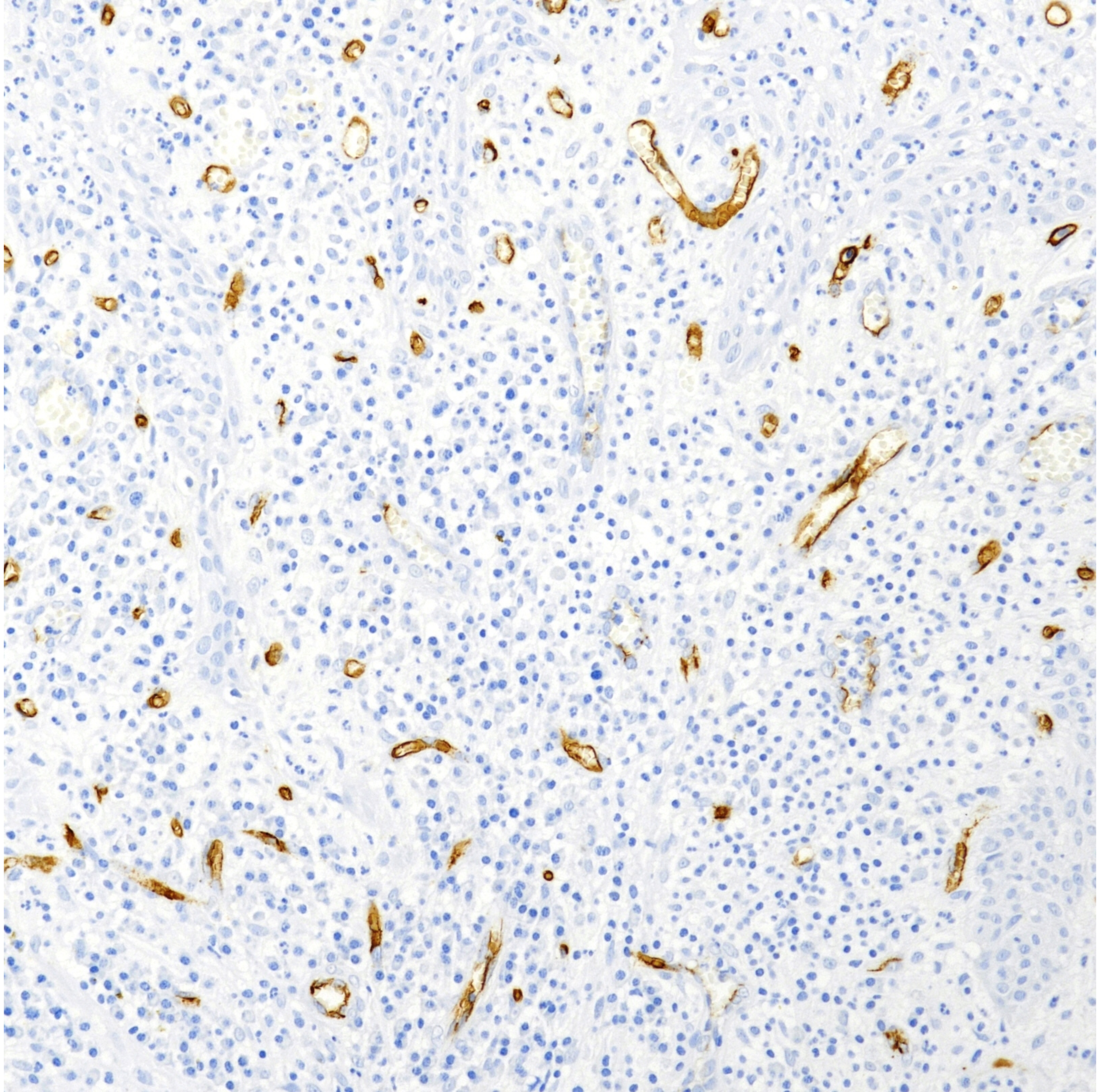

**ID:10 CD34**

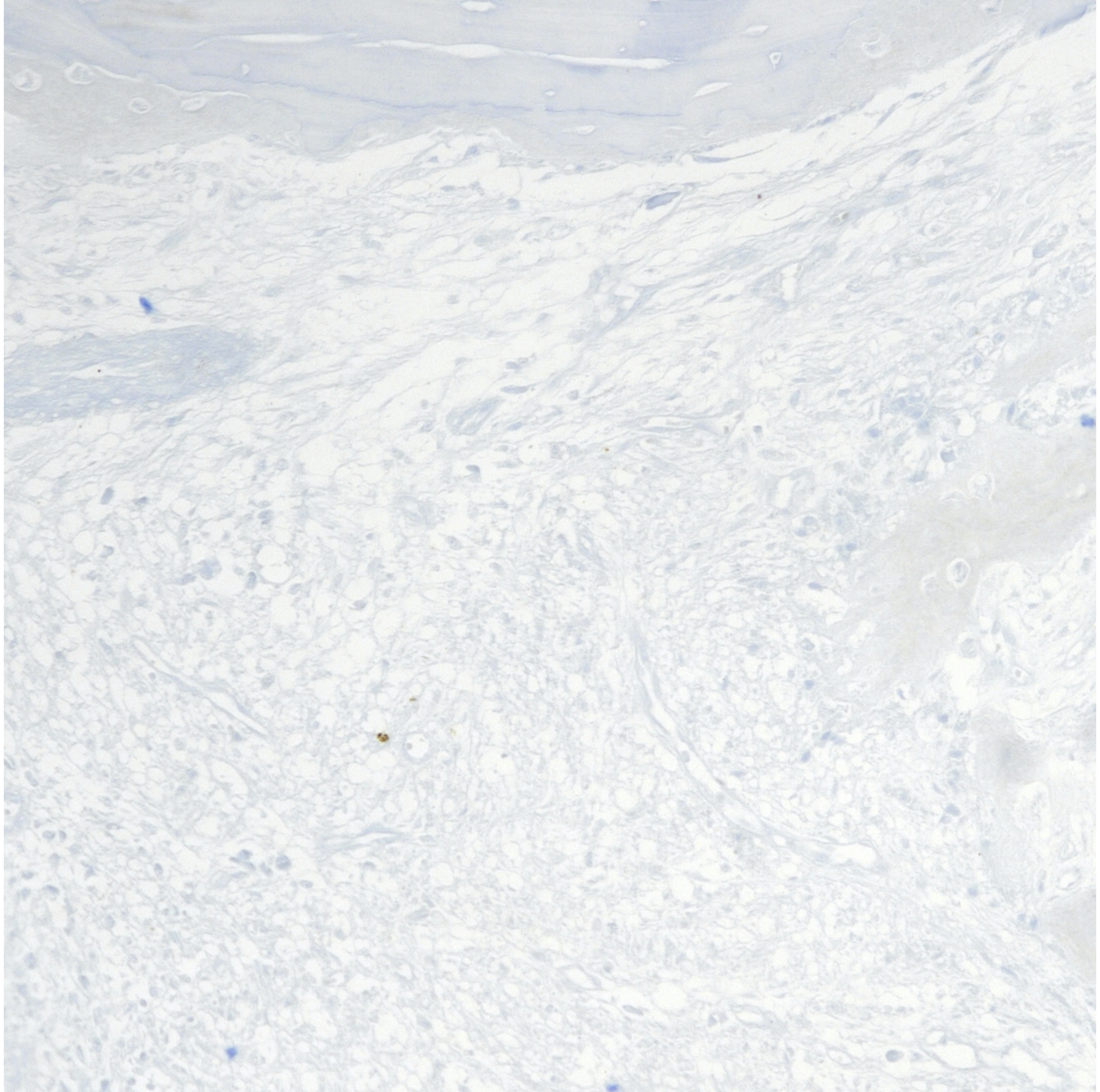

ID:10 CD105

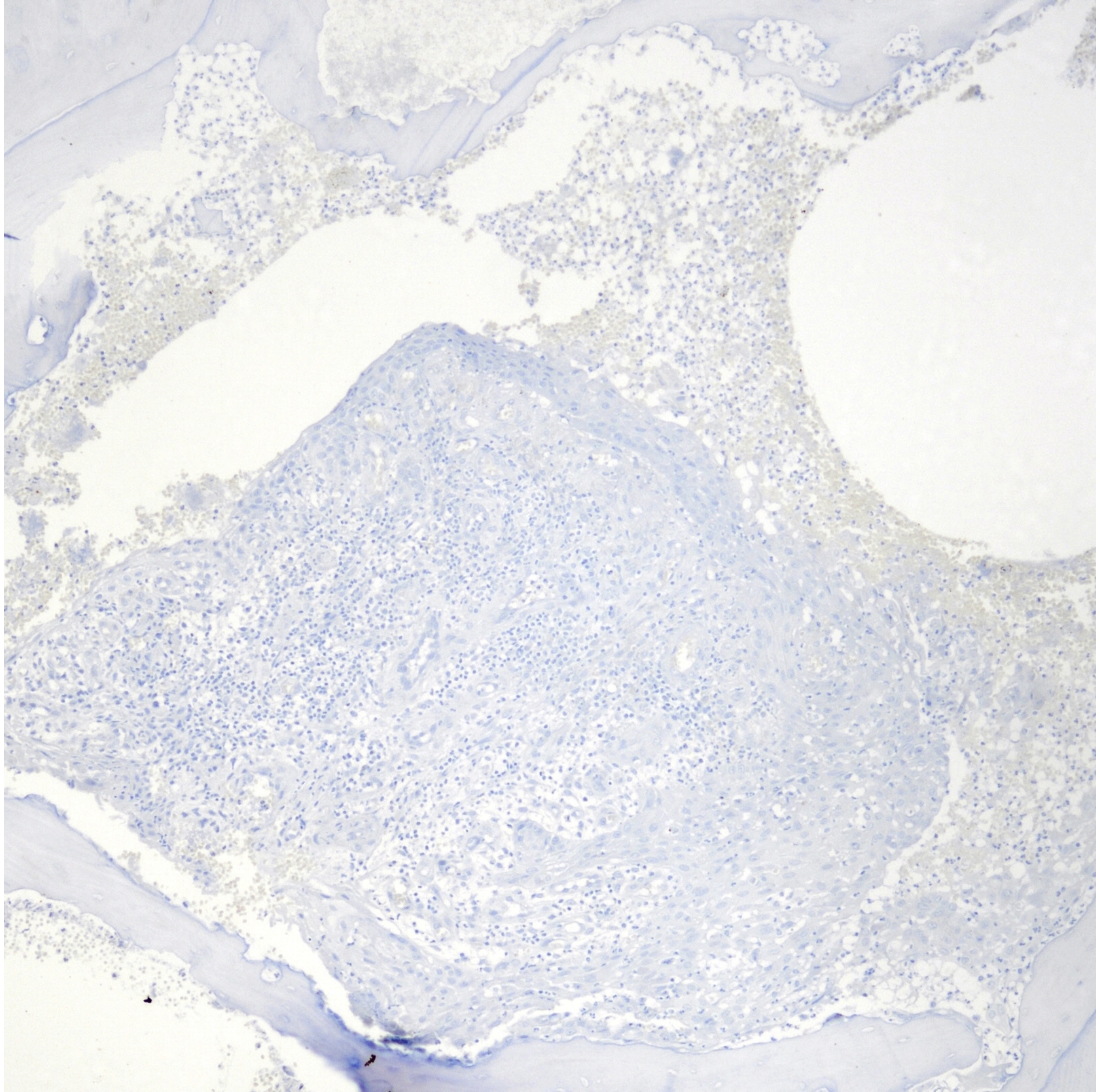

ID:11 CD34

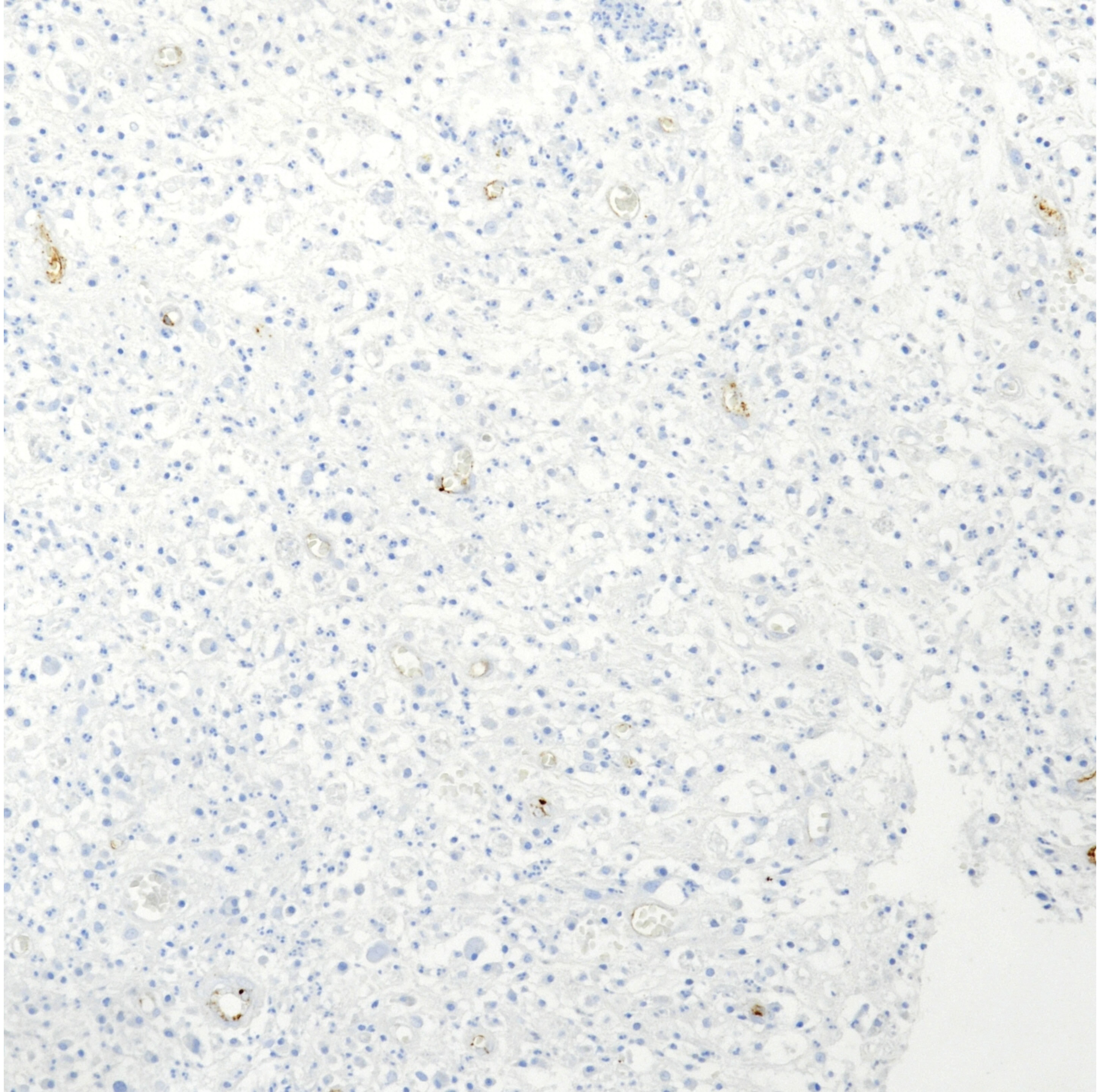

ID:11 CD105

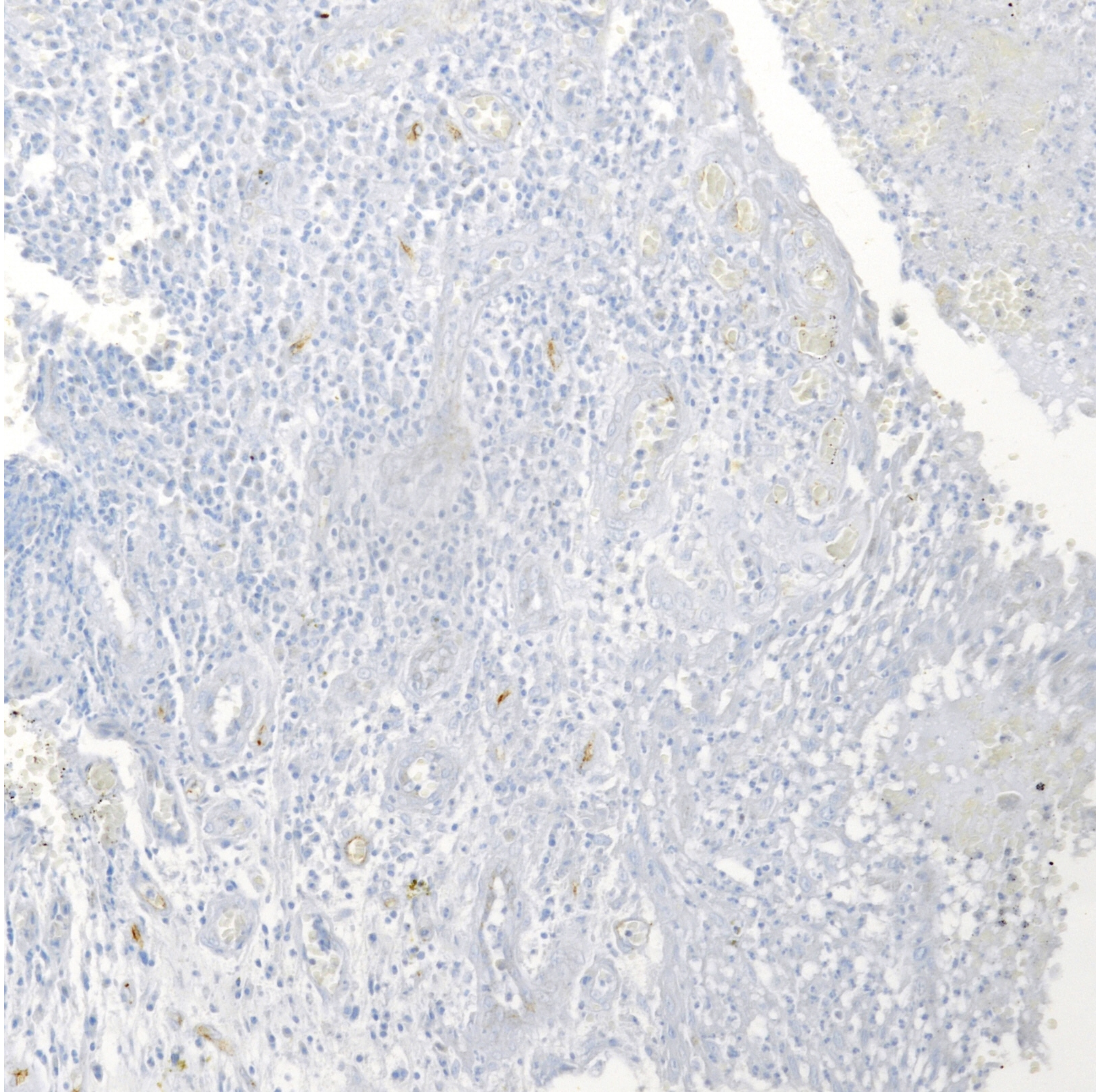

**ID:12  CD34**

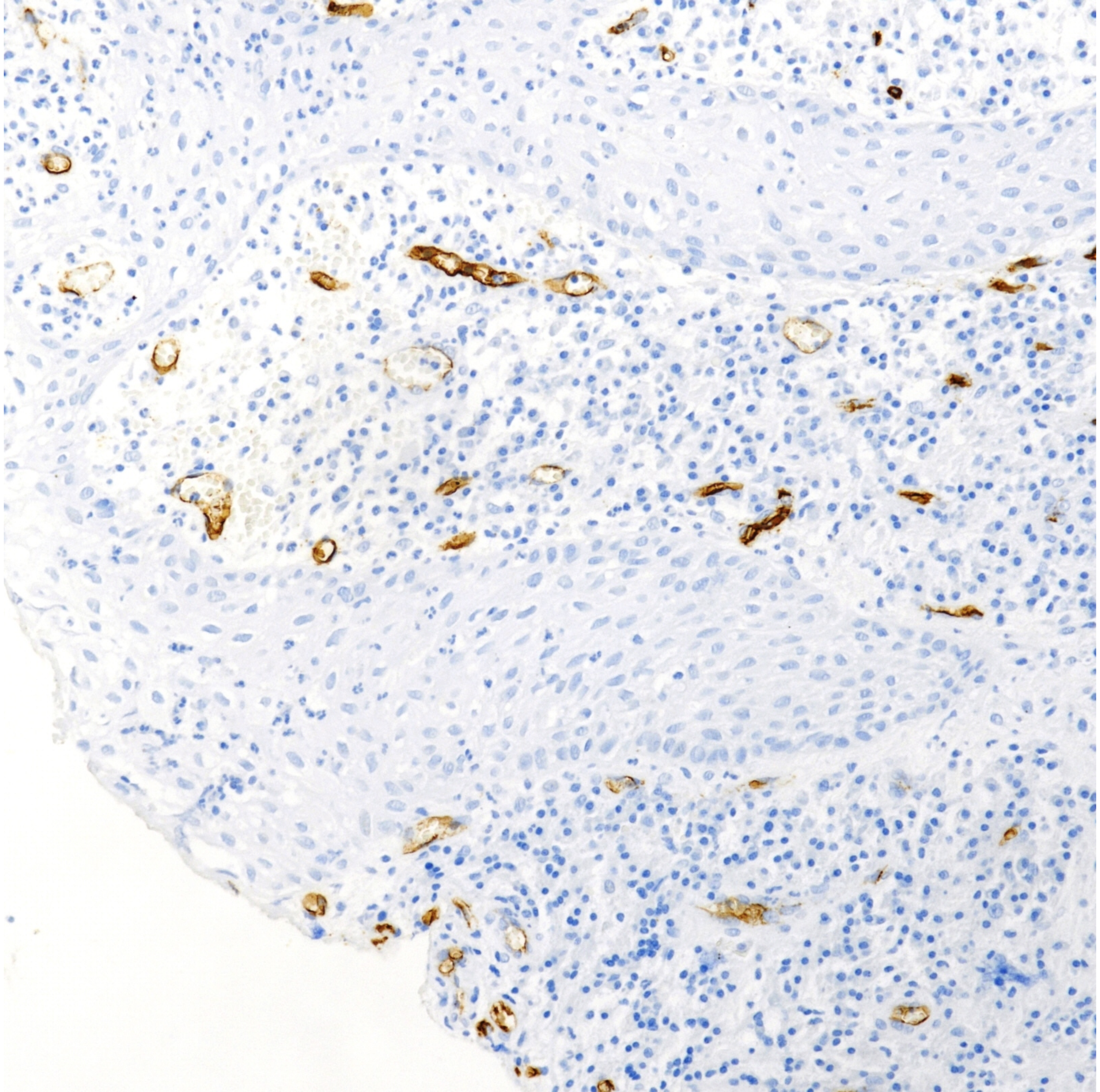

ID:12 CD105

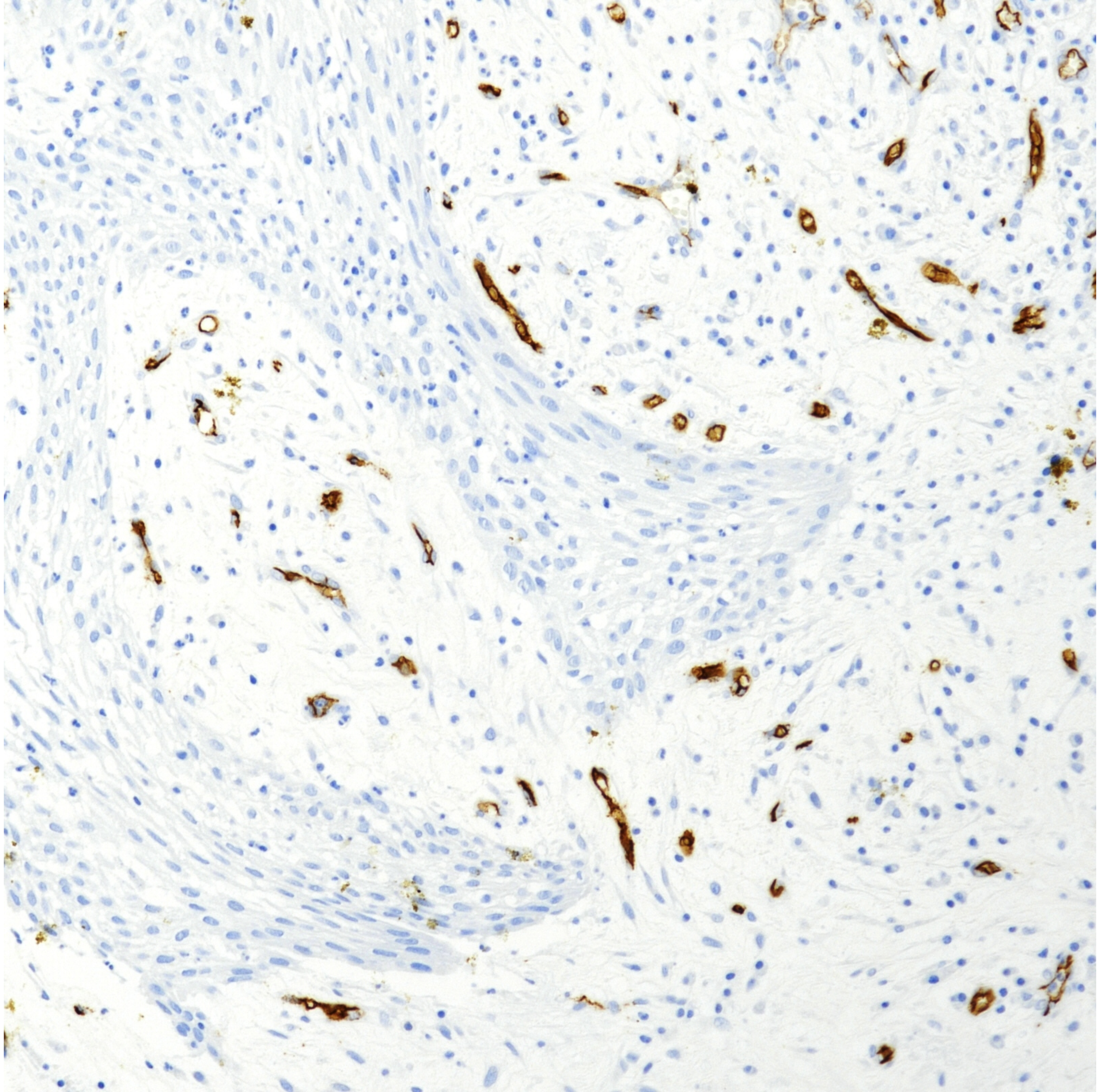

ID:13  CD34

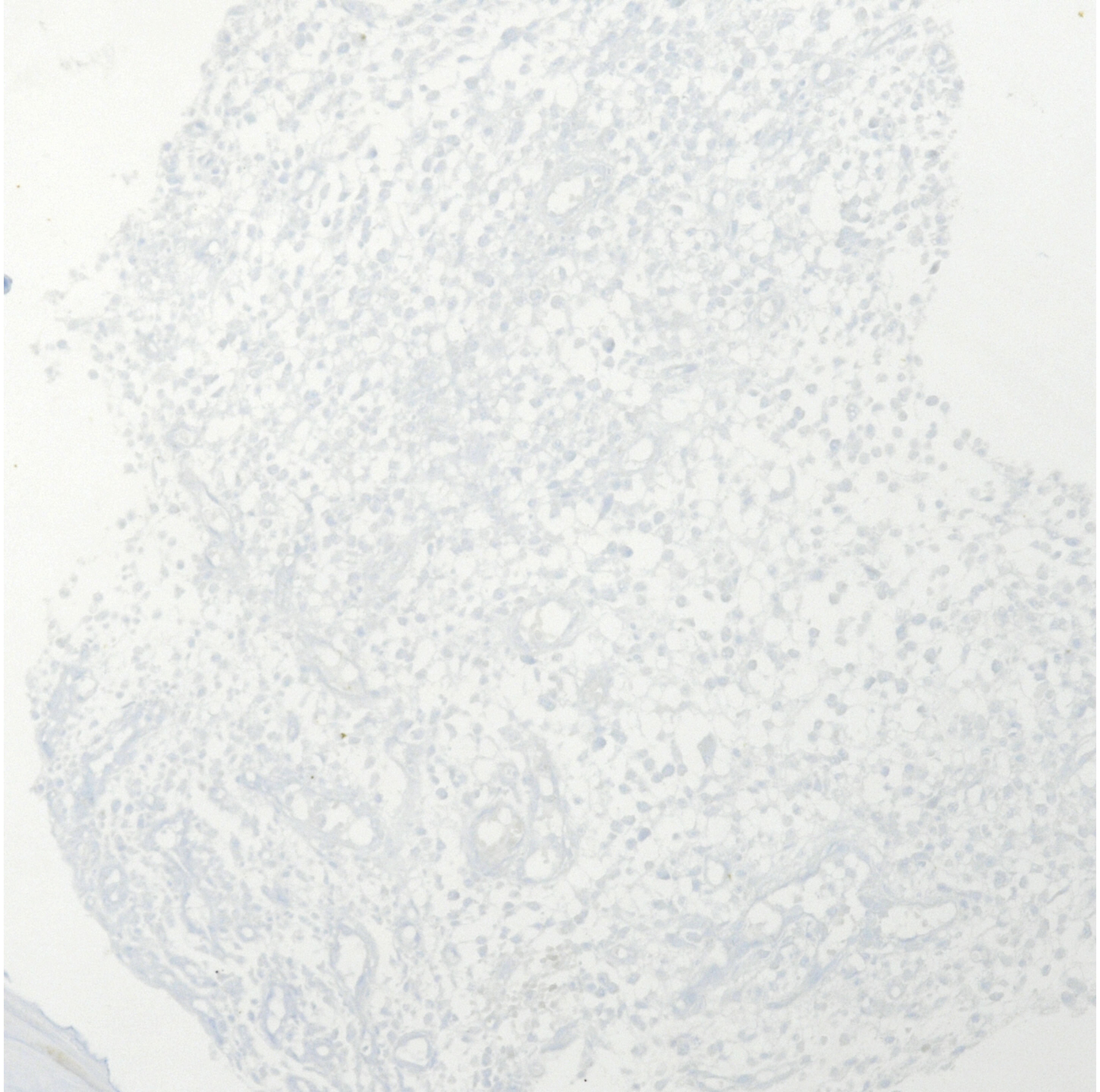

ID:13 CD105

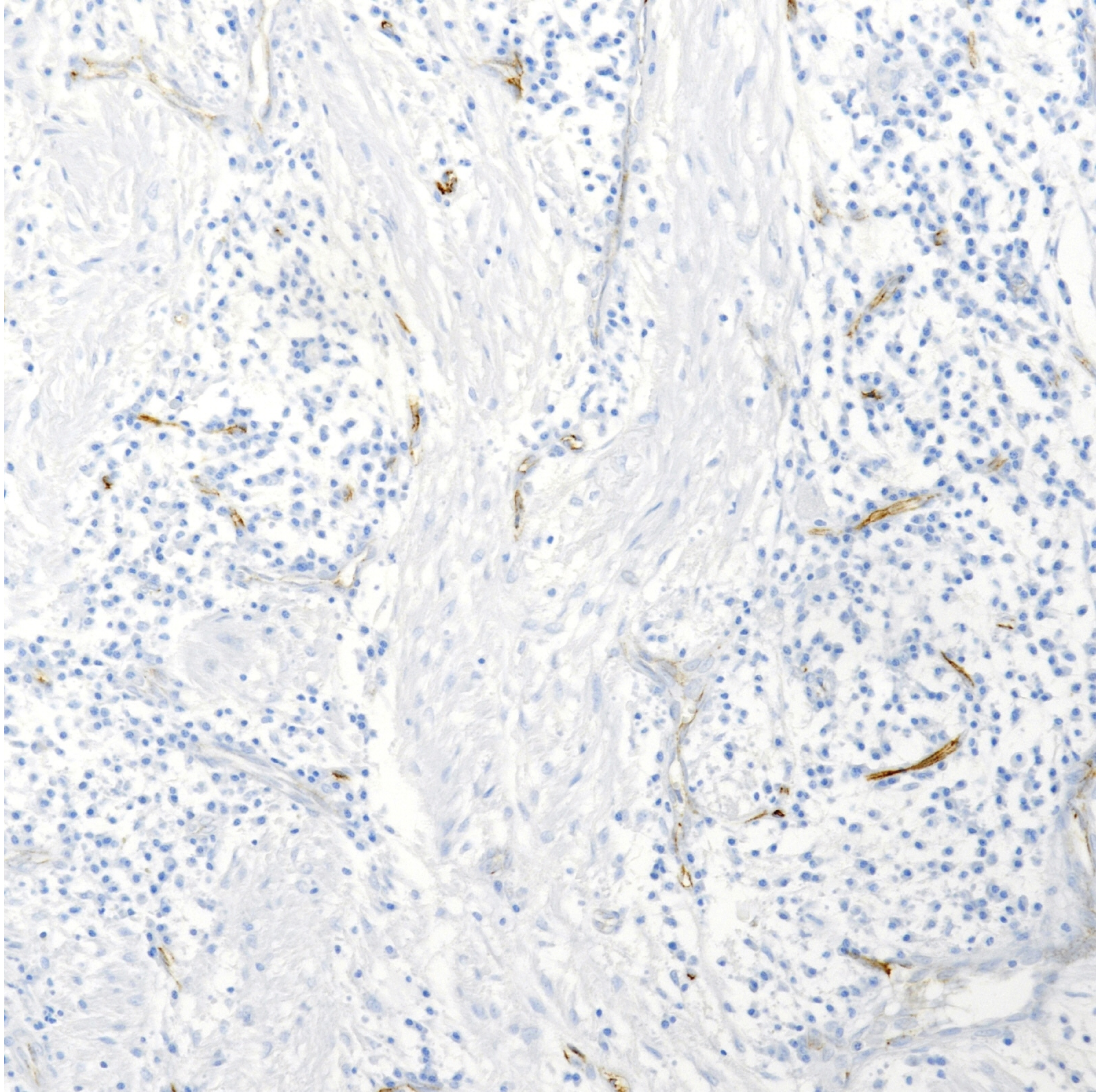

ID:14 CD34

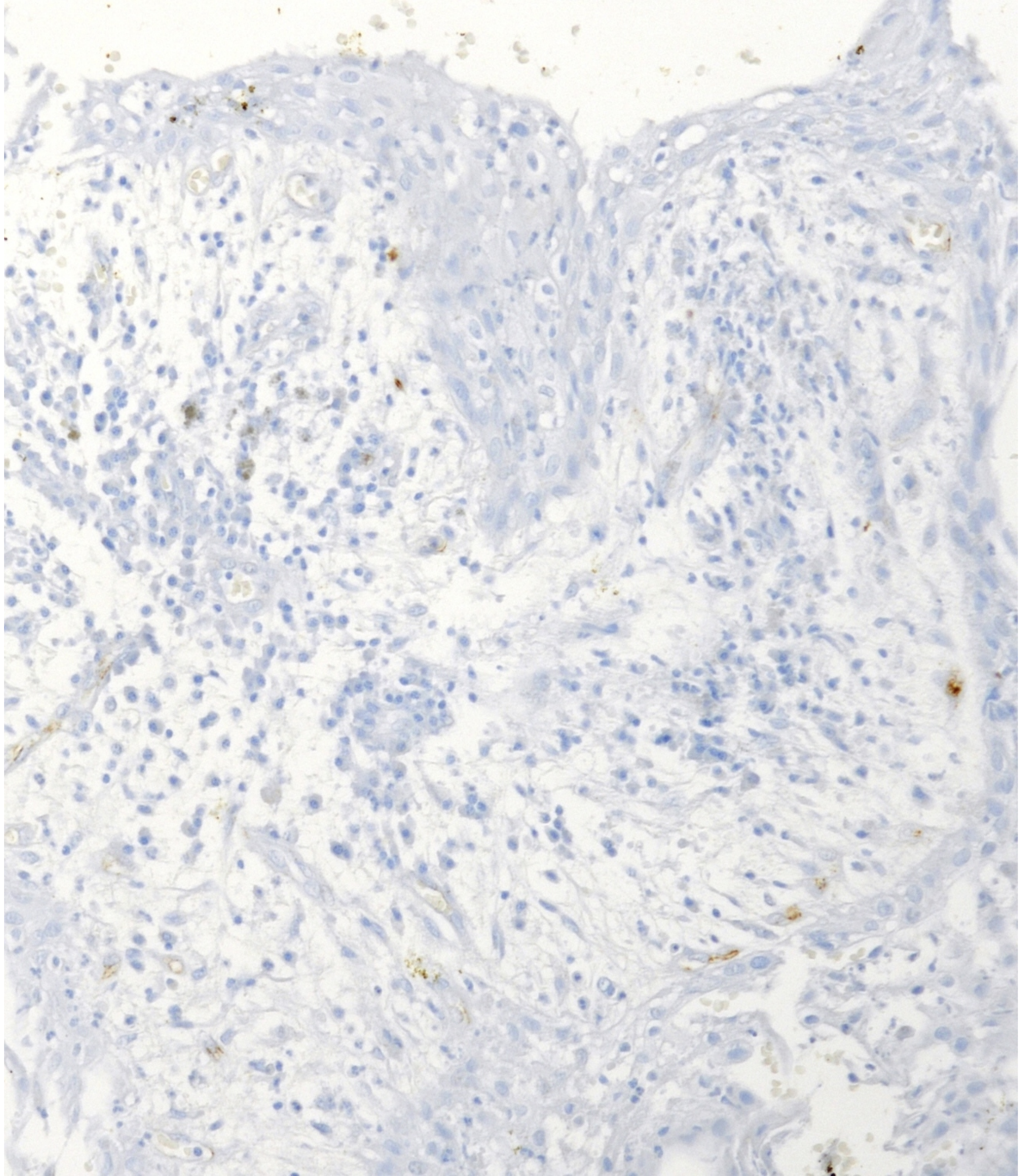

ID:14 CD105

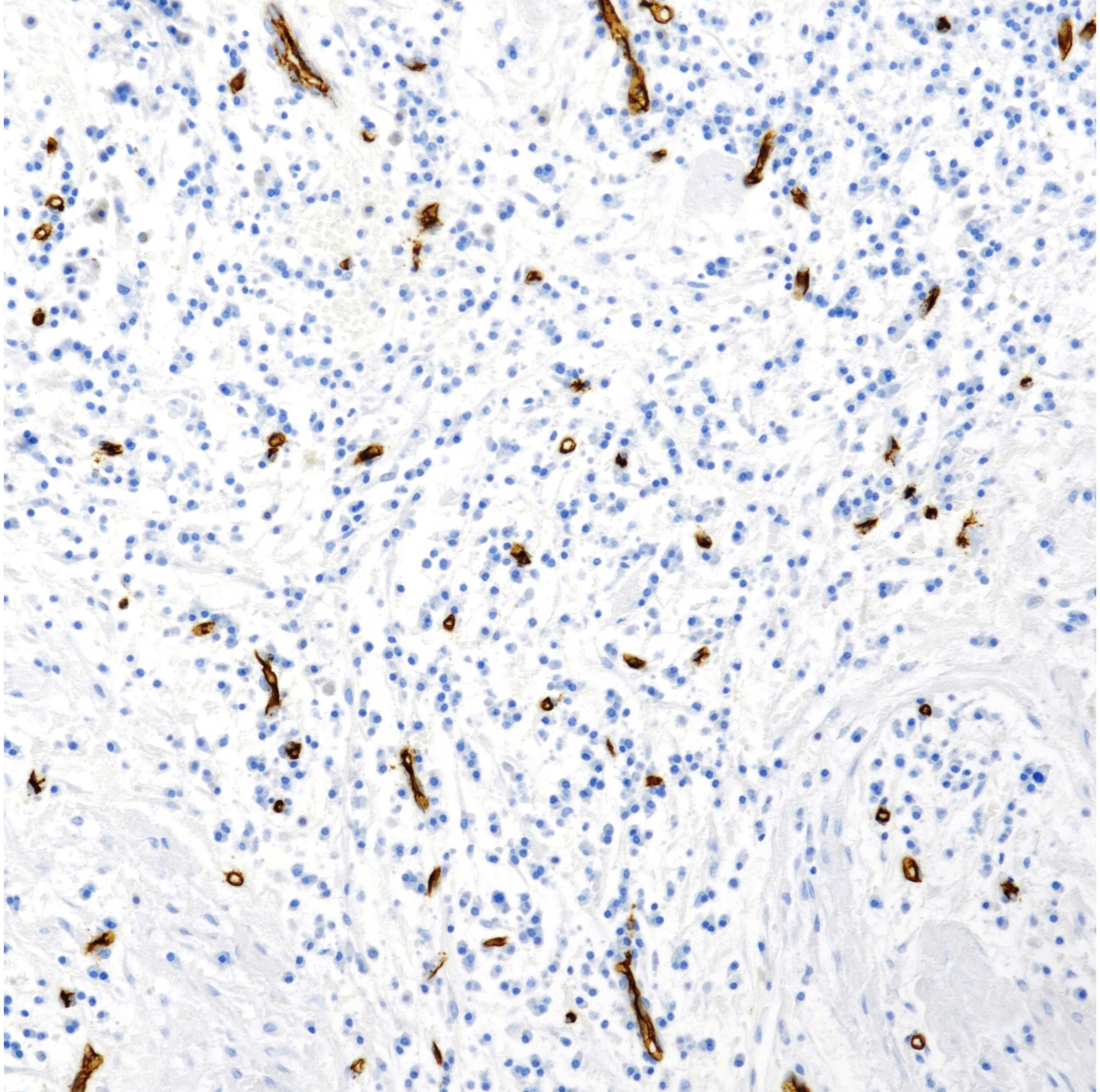

ID:15  CD34

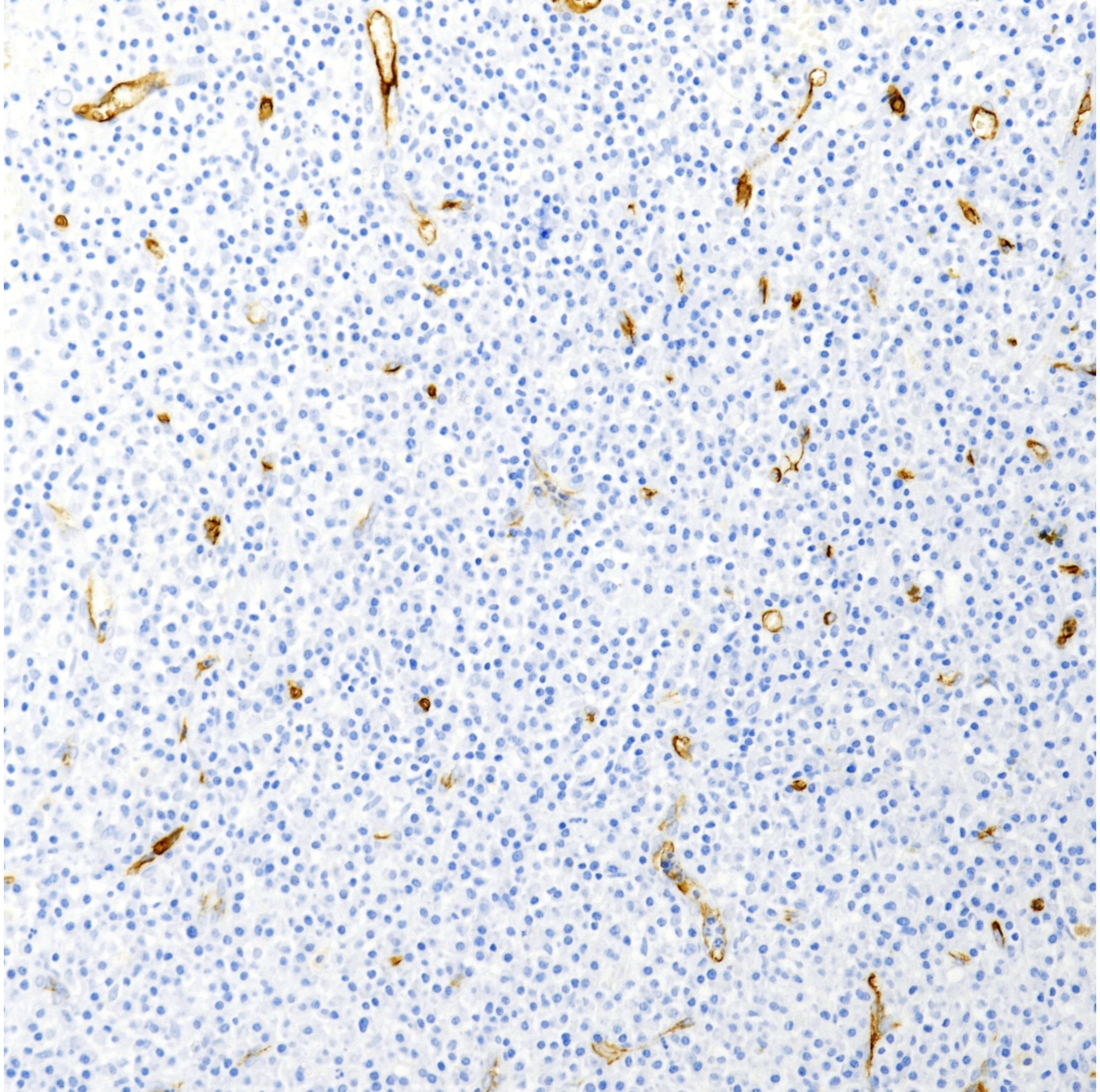

**ID:15 CD105**

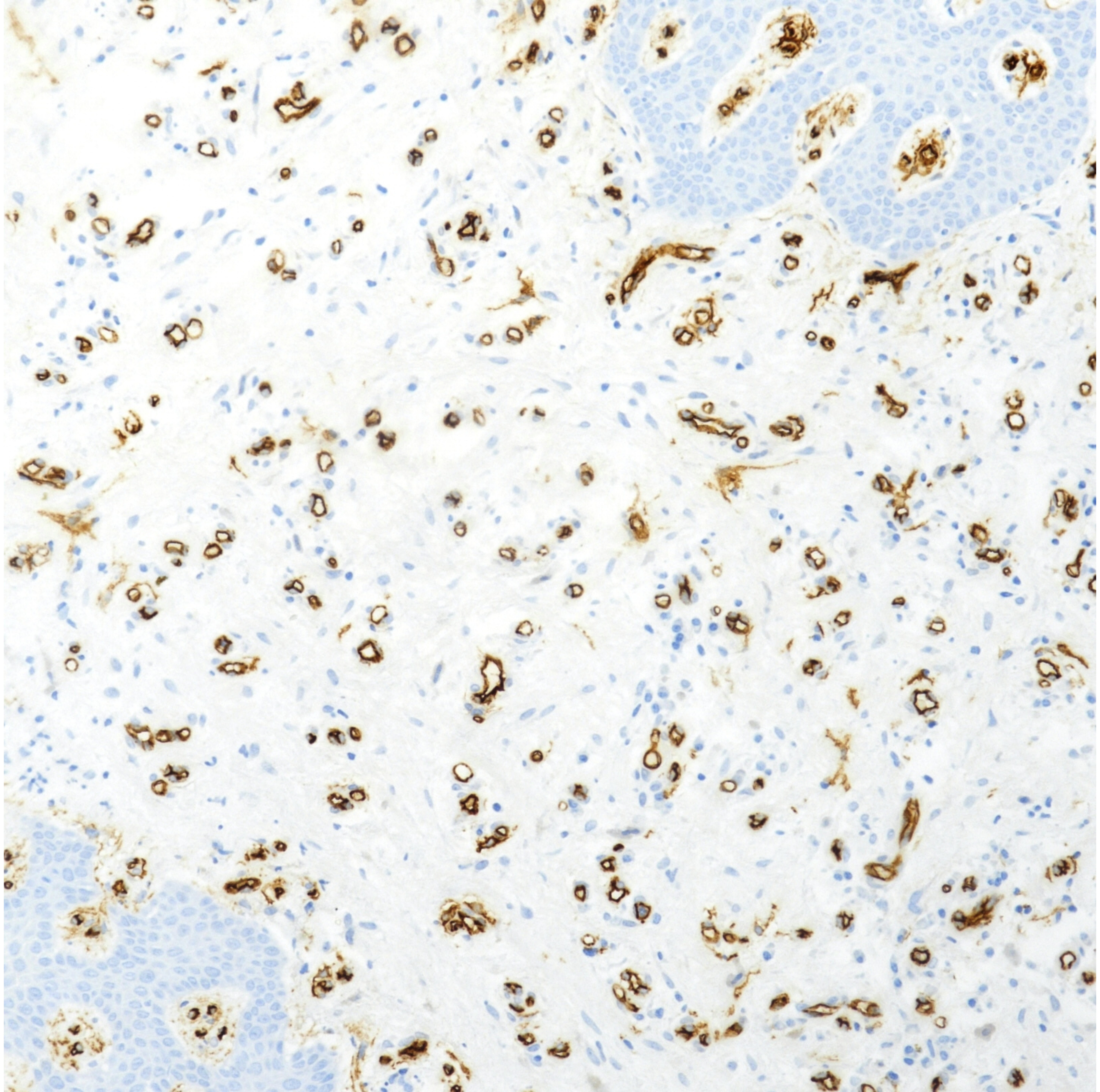

Supplement: Supplementary file 1 [file ijerph-18-11362-s001.zip › ijerph-1390350-supplementary.pdf]
